# Asymmetric Bi and S Single Atoms Over Porous Single‐Crystal TiO_2_ for Efficient CO_2_ Photoreduction to Acetic Acid

**DOI:** 10.1002/adma.202517586

**Published:** 2026-02-18

**Authors:** Guangri Jia, Ying Wang, Mingzi Sun, Yingchuan Zhang, Zhipeng Xie, Xiaoqiang Cui, Bolong Huang, Jimmy C. Yu, Zhengxiao Guo

**Affiliations:** ^1^ Department of Chemistry and HKU‐CAS Joint Laboratory On New Materials The University of Hong Kong Hong Kong Hong Kong SAR; ^2^ Key Laboratory of Superlight Materials and Surface Technology Ministry of Education College of Materials Science and Chemical Engineering Harbin Engineering University Harbin P. R. China; ^3^ Department of Chemistry City University of Hong Kong Kowloon Hong Kong SAR; ^4^ State Key Laboratory of Automotive Simulation and Control, School of Materials Science and Engineering, Key Laboratory of Automobile Materials of MOE Jilin University Changchun P. R. China; ^5^ Department of Chemistry The Chinese University of Hong Kong Hong Kong Hong Kong SAR

**Keywords:** CO_2_ reduction reaction, lewis base sites, photoreduction, structure‐activity relationship, triadic synergy

## Abstract

Regulating multi‐step photocatalytic conversion of molecules remains challenging, primarily due to the complex interplays among light absorption, reactant binding, and charge separation and transfer processes. Here, the photocatalytic conversion of CO_2_ to acetic acid is effectively achieved via the triadic synergy of asymmetric Bi (Bi–O_4_), S (S–O_2_), and 3D porous single‐crystal TiO_2_, which is realized through a selective extraction process. Specifically, Bi active sites lower the energy barrier for CHO^*^ generation and C─C coupling; meanwhile, the S─O structure modulates Bi─O and Ti─O configurations to form strong Lewis base site ((SO_2–_BiO_4_)^δ−^) by constructing a surface sulfate species, thereby accelerating the hydrogenation step in CO_2_ reduction. The specifically designed photocatalytic system achieves a high acetic acid production rate of 66.7 µmol g^−1^ h^−1^ with over 89% selectivity. This design underscores the significance of engineering synergistic active sites and charge transfer to enhance photocatalytic conversion efficiency, offering valuable insight into the structure‐activity relationship for developing high‐performance photocatalysts.

## Graphical abstract

Photocatalytic CO_2_ to acetic acid is achieved over triadic synergy among asymmetric Bi–O_4_, S–O_2_ and 3D porous single‐crystal TiO_2,_ harnessed by a selective extraction process. Particularly, the Bi sites lower the energy barrier for CHO^*^ and C─C coupling, and the structure of S─O regulates Bi─O and Ti─O as a strong Lewis base site ((SO_2_–BiO_4_)^δ−^) to accelerate hydrogenation in CO_2_ reduction.

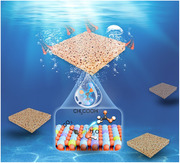

## 1 Introduction

Photocatalytic CO_2_ reduction reaction (CO_2_RR) represents a transformative carbon‐neutral technology, utilizing solar energy to convert CO_2_ into sustainable fuels and industrial feedstocks. This process thereby establishes a green pathway toward net‐negative emissions through renewable carbon cycling. A key challenge lies in engineering the transformation of C═O bonds, as the reduction process involves multiple electron and proton transfers to form a specific target product [1, 2, 3]. This challenge is even more pronounced in photocatalysis, which requires not only the precise control of the catalytic reactions but also the effective generation, separation, and transfer of photogenerated charges [4, 5]. Such complexity is further amplified when the conversion targets C_2+_ product (e.g., acetic acid) as a high‐value industrial commodity [6]. Integrating critical properties including efficient active sites, extended light absorption, and efficient charge transfer into a single photocatalytic system remains a significant challenge [7, 8, 9]. Therefore, constructing a microenvironment with well‐defined functional roles would greatly facilitate the implementation of complex photocatalytic CO_2_ reduction processes.

Synergistic effects of catalytic active sites have recently garnered significant attention due to complexity of catalytic reactions and the elusiveness of reaction intermediates [10, 11, 12]. For complex photocatalytic molecular transformations, the coupling of multiple functionally integrated sites with substrates substantially enhances the typically low conversion efficiency [13]. For instance, introducing pronounced p–d orbital hybridization between different active sites and the substrate not only regulates electron transfer during the transformation of catalytic reaction intermediates and modulates the band structure to extend the light response range [14, 15], but also creates point defects that facilitate the adsorption and bonding of molecules—an effect exemplified in CO_2_ conversion processes, where the key intermediate CO^*^ is effectively activated [16, 17, 18, 19, 20]. Furthermore, the polarization of coupled sites enables effective regulation of electronic structure around the active sites, allowing them to function as frustrated Lewis (acid and base) pairs [21, 22].

Single‐crystal materials exhibit high efficiency in transferring photogenerated carriers to surfaces and interfaces due to much reduced scattering by disordered atomic arrangements [23, 24]. However, traditional synthesis methods for single‐crystal materials often lead to limited specific surface areas, thereby resulting in a low density of catalytically active sites [25]. The construction of porous single‐crystal structures provides a potential solution, which not only optimizes electronic properties but also increases active site density, thus overcoming the inherent challenges associated with charge carrier migration and transfer in conventional single‐crystal materials.

Herein, we report an innovative topological transformation strategy for fabricating a porous single‐crystal TiO_2_ structure, featuring in‐situ formed Bi single‐atom species and concurrently anchored S atomic sites, via the sulfidation/acidation treatment of a Bi_4_Ti_3_O_12_ precursor. This synthetic approach preserves the original structural volume while introducing heterogeneous Bi and S atomic sites onto the single‐crystal TiO_2_ matrix [26]. The resulting (Bi, S)‐codoped TiO_2_ ((Bi, S)TiO_2_) photocatalyst enables efficient CO_2_ selective conversion to acetic acid, which originates from the synergy of multiple electronic and structural effects: first, the hybridization of Bi‐5*p* orbitals with Ti‐3*d* orbitals enhances charge‐carrier separation efficiency and boosts the surface catalytic activity of (Bi, S)TiO_2_; second, the coupling of S‐3*p* orbitals with the O‐2*p* and Ti‐3*d* facilitates interfacial electron transfer and narrows the bandgap. Moreover, the incorporation of S atomic sites (coordinated with two O atoms, denoted as S–O_2_) and Bi atomic sites (coordinated with four O atoms, denoted as Bi–O_4_) into Ti─O frameworks generates sulfated surface moieties, inducing a polarization effect on the metal–oxygen structure (e.g., forming (SO_2–_BiO_4_)^δ−^ species) that acts as a frustrated polarized atomic pair. These integrated functionalities synergistically accelerate the hydrogenation kinetics during CO_2_ reduction by polarizing the adsorbed reactant molecules and stabilizing key reaction intermediates. Consequently, the (Bi, S)TiO_2_ photocatalyst achieves an acetic acid production rate of 66.7 µmol h^−1^ g^−1^ with over 89% selectivity, and exhibits negligible activity degradation even after a 50‐h durability test. This groundbreaking finding demonstrates the successful in‐situ integration of asymmetric frustrated polarized atomic pairs onto 3D porous single‐crystal semiconductors by means of topological transformation, that paves the way for designing high‐performance catalysts for demanding heterogeneous catalytic reactions.

## 2 Results and Discussion

### 2.1 Material Synthesis and Characterizations

**FIGURE 1:**
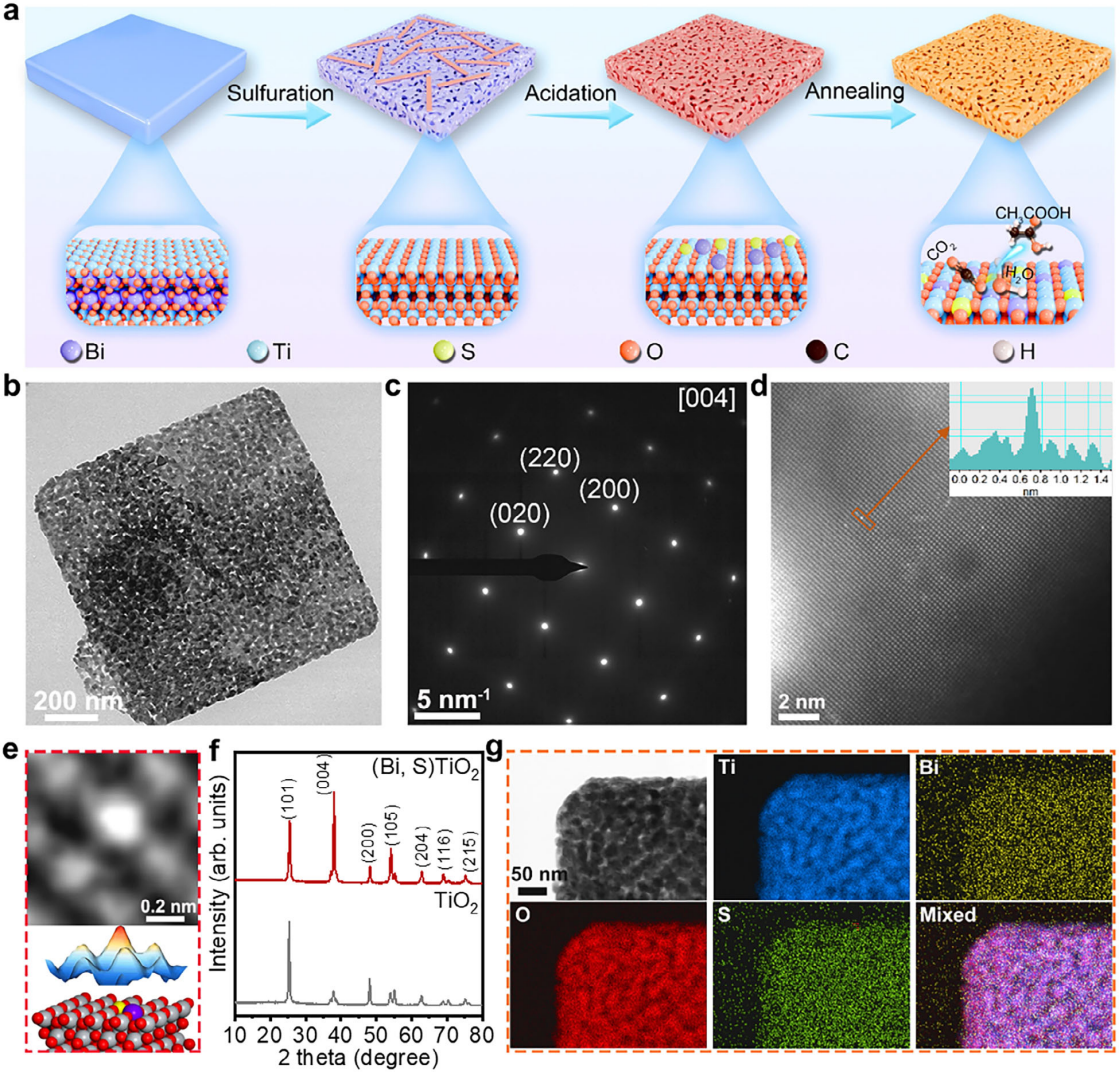
(a) The synthesis process of (Bi, S)TiO_2_. (b) TEM image of (Bi, S)TiO_2_. (c) SAED pattern of (Bi, S)TiO_2_. (d) Cs‐corrected HAADF‐STEM image of (Bi, S)TiO_2_. (e) Enlarged HAADF‐STEM image and corresponding atomistic simulation in (d). (f) XRD pattern of (Bi, S)TiO_2_ and TiO_2_. (g) TEM‐EDS elemental mapping of (Bi, S)TiO_2_.

Using a conventional solid‐state synthesis method (details in Experimental Section of Supporting Information), 2D single‐crystal Bi_4_Ti_3_O_12_ was first synthesized and employed as the preform for photocatalyst fabrication (Figures S1–S4). A hydrothermal treatment conducted under ambient air atmosphere enabled the preferential sulfidation of Bi species within the Bi_4_Ti_3_O_12_ crystal structure. This selective sulfidation behaviour can be attributed to the inherent differences in chemical stability among the various constituent components of the parent Bi_4_Ti_3_O_12_ matrix [27, 28]. This process gradually led to the formation of Bi_2_S_3_ and a porous TiO_2_ matrix structure, as evidenced by the changes in the chemical microenvironment when compared to the non‐sulfidated counterpart (Figures S5–S8). Subsequent removal of the Bi component by acidation, culminating in the creation of a 3D porous single‐crystal TiO_2_ structure. Through careful control of this transformation, atomic‐level Bi and S species can be retained over the TiO_2_ surface and coordinated with O, forming a single‐crystal TiO_2_ structure with embedded Bi and S single atoms (SAs) (Figure 1a; Figures S9).

Characterizations of the synthesized samples by transmission electron microscopy (TEM) and scanning electron microscopy (SEM) reveal distinct 3D porous characteristics (Figure 1b; Figure S10). High‐resolution TEM (HR‐TEM) and selected area electron diffraction (SAED) analyses demonstrate that the entire 3D structure maintains the attributes of a single crystal (Figure 1c; Figure S11), despite the relatively thin strut size (25 nm) among the pores (Figure S10). Further validation of the presence of Bi SAs in (Bi, S)TiO_2_ was achieved via imaging with Cs‐corrected high‐angle annular dark‐field scanning transmission electron microscopy (HAADF‐STEM), which reveals distinct Bi sites replacing Ti sites in the (Bi, S)TiO_2_ lattice (Figure 1d,e). Moreover, due to the introduction of S, the Ti atoms surrounding Bi present an asymmetric structure (inset of Figure 1d). Inductively coupled plasma optical emission spectrometry (ICP‐OES) measurements show that the contents of Bi and S in (Bi, S)TiO_2_ are 1.0 and 1.4 at.%, respectively (Table S1). The main facets of the (Bi, S)TiO_2_ differ from those of TiO_2_ nanoparticles (Figure S12), with the (004) facet being predominant, associated with its underlying 2D structure (Figure 1f; Figure S13). Additionally, transmission electron microscopy‐energy dispersive X‐ray spectroscopy (TEM‐EDS) elemental mapping confirms the coexistence of Bi and S on the TiO_2_ substrate (Figure 1g). The 3D porous structure possesses a high specific surface area (66.3 m^2^ g^−1^) and accelerate mass transfer, both of which contribute favourably to catalytic reactions (Figure S14).

**FIGURE 2:**
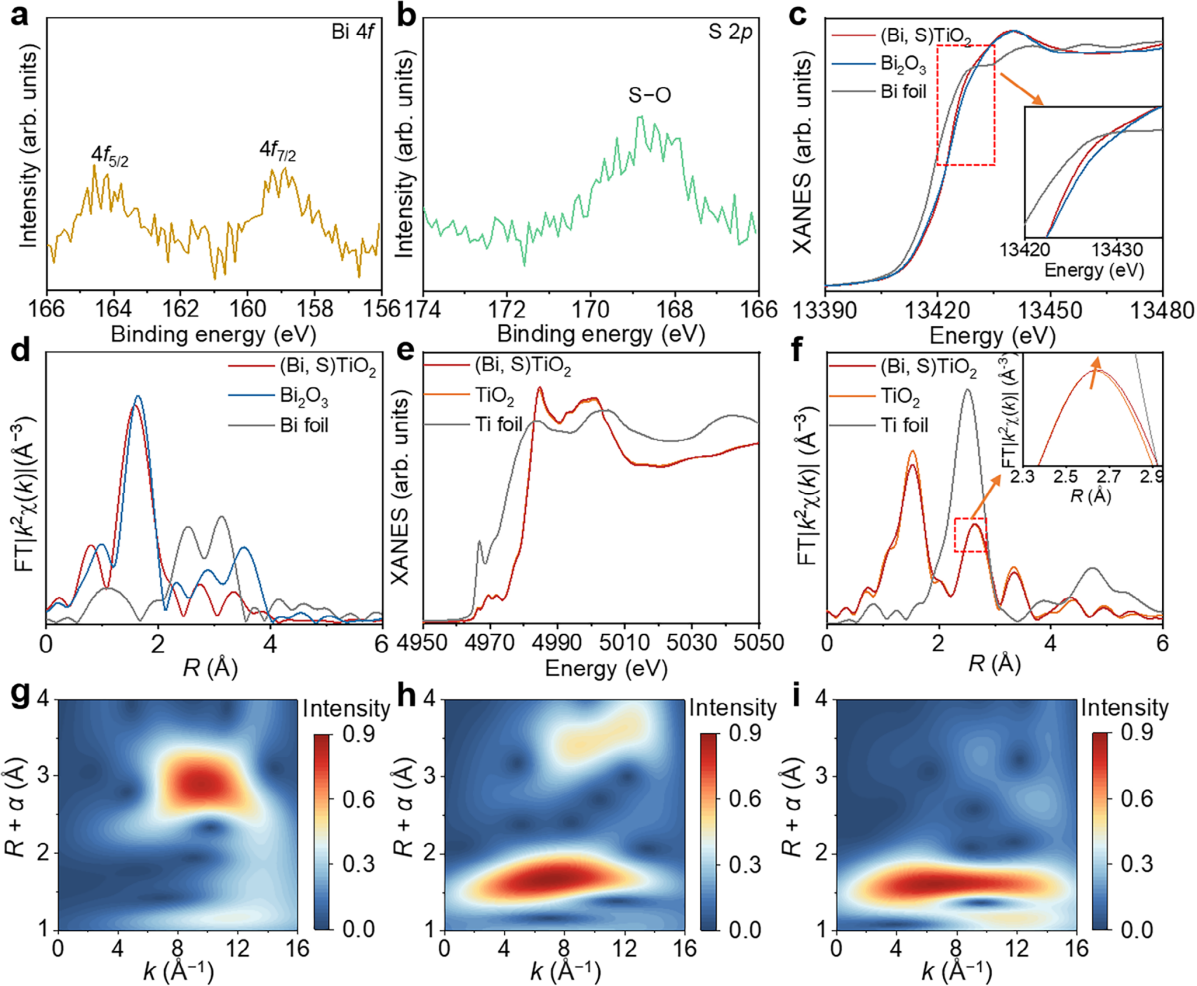
High‐resolution XPS spectra of (a) Bi 4*f* and (b) S 2*p* of (Bi, S)TiO_2_. (c) Bi L‐edge XANES spectra of (Bi, S)TiO_2_, Bi_2_O_3_, and Bi foil. (d) Bi L‐edge FT‐EXAFS spectra of (Bi, S)TiO_2_, Bi_2_O_3_, and Bi foil. (e) Ti K‐edge XANES spectra of (Bi, S)TiO_2_, TiO_2_, and Ti foil. (f) Ti K‐edge FT‐EXAFS spectra of (Bi, S)TiO_2_, TiO_2_, and Ti foil. (g–i) Wavelet transform analysis of *k^2^*‐weighted EXAFS data of Bi foil, Bi_2_O_3_, and (Bi, S)TiO_2_.

Furthermore, the chemical state and coordination environment of the S and Bi species in the (Bi, S)TiO_2_ were analyzed using X‐ray photoelectron spectroscopy (XPS) and X‐ray absorption fine structure (XAFS) techniques. The XPS analysis reveals that Bi predominantly exists in the Bi^3+^ oxidation state, while S is in the form of S─O species (Figure 2a,b; Figure S15a). Further insights into the types and local coordination environments of Bi were obtained through X‐ray absorption spectroscopy (XAS). By comparing the X‐ray absorption near‐edge structure (XANES) of (Bi, S)TiO_2_ with reference materials such as Bi foil and Bi_2_O_3_, it is clear that the oxidation state of Bi in (Bi, S)TiO_2_ is Bi^3+^, consistent with the XPS findings. The XANES spectrum at the Bi L_3_‐edge exhibits a distinct white line peak at 13430 eV, suggesting strong interactions between Bi^3+^ and the TiO_2_ substrate (Figure 2c). In the extended X‐ray absorption fine structure (EXAFS) analysis (Figure 2d), the peak at 3.0 Å in the Bi foil's spectrum corresponds to typical Bi─Bi scattering. In contrast, the EXAFS spectrum of (Bi, S)TiO_2_ shows a peak at 1.63 Å, indicative of a Bi─O signal with no detectable Bi─Bi signal, confirming the presence of Bi sites as SAs in the catalyst. The varying coordination environments of Bi in (Bi, S)TiO_2_ lead to a shift in the Bi─O bond length (1.67 Å) compared to that in Bi_2_O_3_. Furthermore, the oxidation state of S in (Bi, S)TiO_2_ is determined to range from +4 to +6, compared with standard samples of Na_2_SO_3_ and Na_2_SO_4_ (Figure S16a). Additionally, EXAFS analysis reveals the local coordination environment of S atoms, which exist in the form of S─O structures (Figure S16b).

Additionally, multi‐shell EXAFS best‐fit analysis reveals two distinct Bi─O bonds, which is attributed to the Jahn–Teller effect (Figure S17a and Table S2) [29]. Although the valence states are similar (Figure 2e; Figure S15b), the Ti─Ti bond length exhibits an obvious change (from 2.63 to 2.65 Å) due to the incorporation of Bi and S, when compared with pristine TiO_2_ (Figure 2f; Figures S18–S20). Further fitting was performed for S K‐edge, the coordination number of oxidized sulfur species in the (Bi, S)TiO_2_ photocatalyst is significantly lower than that in Na_2_SO_3_ and Na_2_SO_4_, being only 2 (Figure S17b and Table S2) due to the unsaturated nature of surface doping. This low‐coordination characteristics further confirms that sulfur is integrated into the surface lattice rather than existing as freely adsorbed sulfate/sulfite ions, which aligns well with our previous EXAFS observations regarding the S─O bonding environment. Specifically, the under‐coordinated S and Bi active sites introduced by surface doping create a more electron‐rich local chemical environment, which can enhance the electrostatic interaction between the catalyst surface and the electrophilic CO_2_ molecules (characterized by a linear O═C═O structure with partial positive charge on the central C atom). Wavelet transform (WT) analysis enables high‐resolution identification of the coordination environment in *k* space and *R* space. The WT contour plots for Bi foil and Bi_2_O_3_ (Figure 2g,h) show a peak at 10 Å^−1^ in *k* space, which corresponds to the Bi─Bi configuration and is not observed in (Bi, S)TiO_2_. Additionally, the WT spectrum of (Bi, S)TiO_2_ exhibits a widened peak at *k* space when compared to that of Bi_2_O_3_, attributed to the distinct Bi‐coordinated O environment (Bi–O_4_) induced by the introduction of S.

### 2.2 Band Structure and Charge Dynamics

The analysis of the energy band structure and charge separation/transfer behaviour in photocatalysts is crucial for engineering photocatalytic performance. The introduction of the hetero‐elements leads to the formation of appropriate intermediate bandgaps, which enhances light absorption in the visible range (Figure S21). Further characterization using ultraviolet photoelectron spectroscopy (UPS) confirms the existence of distinct valence band positions in (Bi, S)TiO_2_ when compared with pristine TiO_2_ (Figure S22). When combined with the bandgap, it is clear that the conduction band is more favourable for photocatalytic reduction of CO_2_ (Table S3).

**FIGURE 3:**
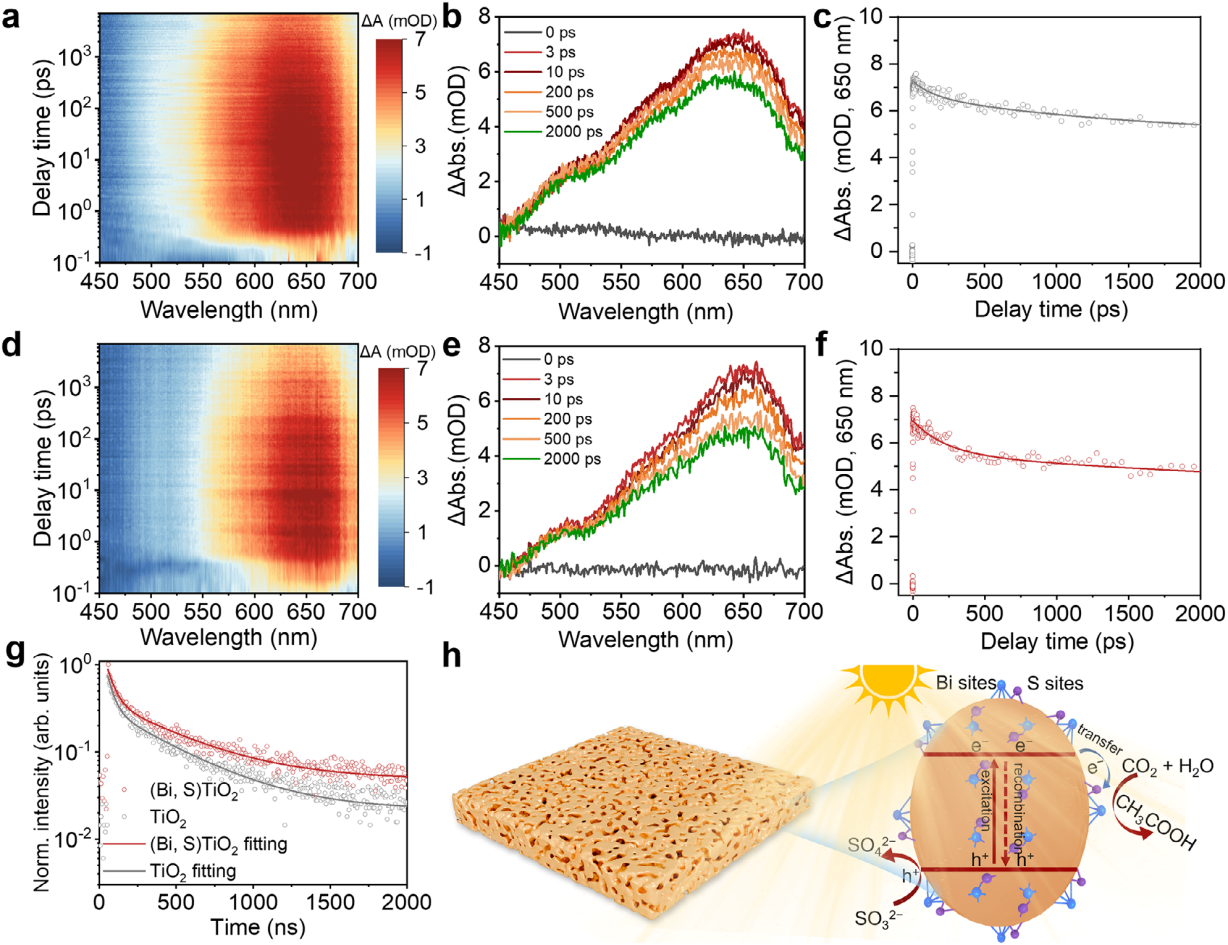
TA spectra and carriers’ kinetics of (a–c) TiO_2_ and (d–f) (Bi, S)TiO_2_ under 360 nm pump laser excitation. (g) TRPL spectra of TiO_2_ and (Bi, S)TiO_2_. (h) Scheme of dynamics of photogenerated charges for (Bi, S)TiO_2_.

The dynamics and lifetime of photoexcited carriers are critical for photocatalytic reactions. To investigate this, we employed femtosecond‐transient absorption (fs‐TA) spectroscopy to compare the ultrafast exciton dynamics of TiO_2_ and (Bi, S)TiO_2_. Based on the light absorption spectra of the samples, 360 nm was selected as the pump wavelength. Notably, both samples exhibit broad positive absorption bands in the continuous probe wavelength range of 550–700 nm, with a maximum absorption peak at ∼650 nm, which is attributed to excited state absorption (Figure 3a,d). We recorded the transient attenuation curves of TiO_2_ and (Bi, S)TiO_2_ at 650 nm and fitted them using a double‐exponential function to analyse the dynamics of photogenerated electrons (Figure 3c–f). Here, the short time (τ_1_) and the long time (τ_2_) correspond to the photogenerated electrons trapped by the shallow and the deep states, respectively. Owing to the bonding structure of Bi─O─Ti, charges can be effectively trapped at the interface between the Bi─O moiety and the bulk Ti─O framework. This effect strongly delays the charge self‐trapping process in the TiO_2_ component and further suppresses the recombination of active charge carriers [30]. The long lifetime (τ_2_) of (Bi, S)TiO_2_ is 4538 ps, which is substantially longer than that of TiO_2_ (τ_2_ = 1686.2 ps, Table S4). This enhancement is primarily attributed to two factors: first, single‐crystal TiO_2_ facilitates carrier transport to the crystal surface; second, the charge transfer between the Bi─O single‐atom structure and TiO_2_ strongly delays the charge recombination process in TiO_2_ components, thereby prolonging the lifetime of active charge carriers.

In addition, the separation and recombination of photoelectron–hole pairs were investigated via photoluminescence (PL) spectroscopy. Pristine TiO_2_ shows strong luminescence at 550 nm, whereas the PL intensity of (Bi, S)TiO_2_ is nearly quenched due to the efficient charge transfer (Figure S23). Corresponding time‐resolved PL decay profiles under 360 nm excitation were also recorded and fitted using double‐exponential decay dynamics. Compared with pristine TiO_2_, (Bi, S)TiO_2_ shows a significant increase in PL lifetime, reaching 509 ns (Figure 3g and Table S5). Furthermore, electrochemical impedance spectrum (EIS) and photocurrent measurements reveal that (Bi, S)TiO_2_ exhibits lower charge‐transfer impedance and a higher photocurrent response than pristine TiO_2_ (Figure S24). These observations can be rationalized based on structural features of (Bi, S)TiO_2_: notably, the presence of single‐atom sites provides abundant carrier transfer channels, minimizing energy dissipation during long‐distance transport. Single‐atom sites form strong electronic interactions with TiO_2_ substrates via high dispersion and unsaturated coordination, creating localized interface states that selectively trap photogenerated electrons/holes. Acting as “temporary storage”, these single‐atom sites facilitate the rapid transfer of trapped carriers to reactants, thereby reducing electron‐hole recombination.

Meanwhile, benefiting from the single‐crystal nature of the substrate, the internal atoms are arranged regularly, eliminating hindrances by grain boundaries or grain boundary defects. This structural advantage enables photogenerated electron‐hole pairs to migrate directionally along the crystal lattice, avoiding carrier scattering and stagnation caused by grain boundaries in polycrystalline materials. Additionally, the single‐crystal structure minimizes defects (e.g., dislocations and vacancies) that act as major charge recombination centers, resulting in a significantly lower carrier recombination rate compared with polycrystalline counterparts. Collectively, these effects greatly promote the subsequent charge transfer to the catalyst surface for photocatalytic reactions (Figure 3h).

### 2.3 Photocatalytic CO_2_RR Performance

**FIGURE 4:**
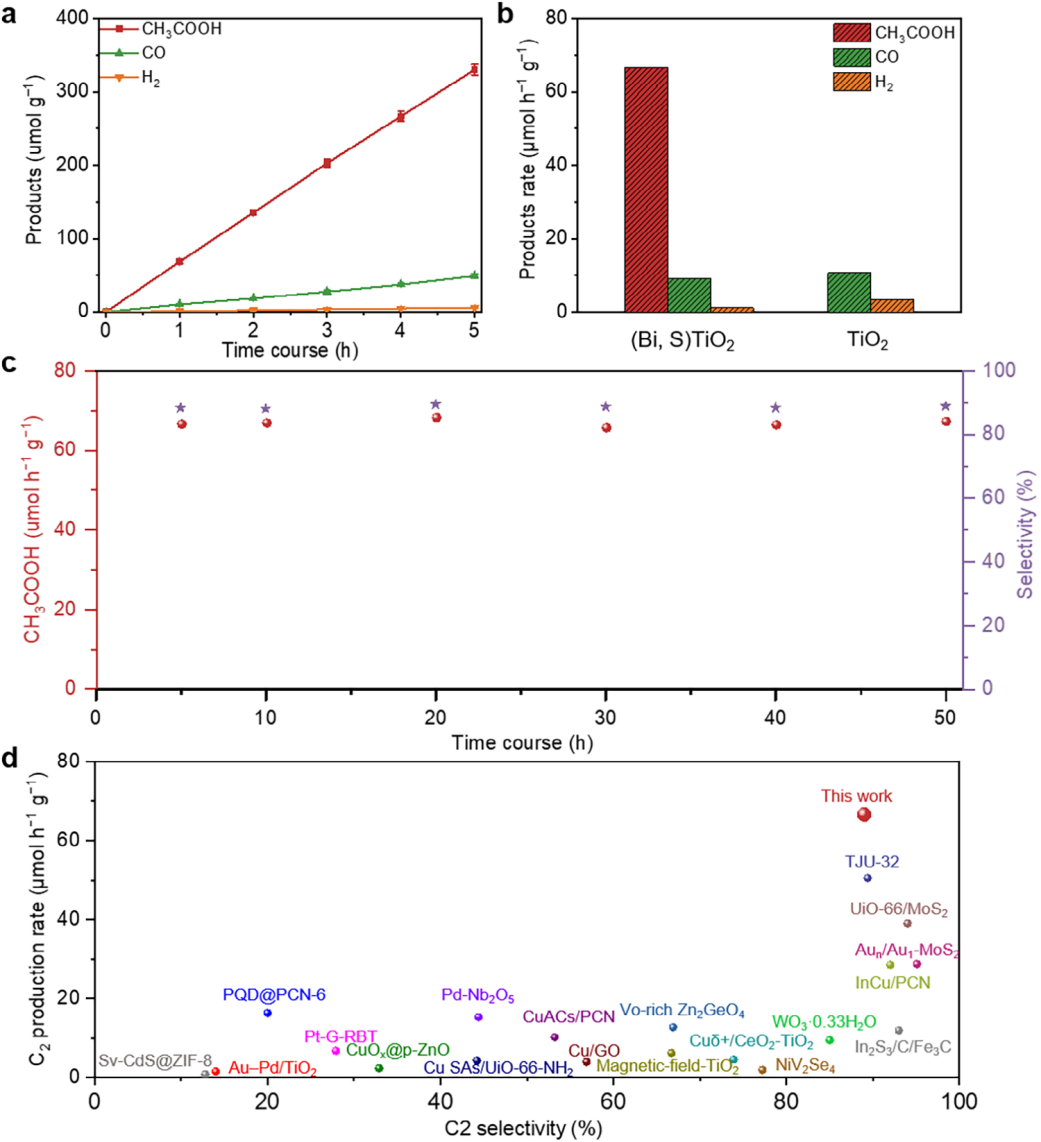
(a) Time course of photocatalytic CO_2_RR for (Bi, S)TiO_2_. (b) Rates of photocatalytic product generation for (Bi, S)TiO_2_ and TiO_2_. (c) Longtime measurements and selectivity of photocatalytic CO_2_RR for (Bi, S)TiO_2_. (d) Comparison of produced C_2_ product rate and selectivity with those of reported photocatalysts.

Leveraging the structural insights into (Bi, S)TiO_2_, we evaluated its photocatalytic CO_2_ reduction performance. Under full‐spectrum illumination, ^1^H nuclear magnetic resonance (NMR) and gas chromatography analyses confirm that (Bi, S)TiO_2_ produces H_2_, CO, CH_3_COOH at rates of 1.2, 9.2, and 66.7 µmol g^−1^ h^−1^, respectively, with product yields showing consistent linear growth over a specified time period (Figure 4a; Figure S25). The apparent quantum yields are 0.124% at 350 nm and 0.083% at 380 nm for CO_2_RR to CH_3_COOH. In contrast, pristine TiO_2_, lacking active Bi and S sites, fails to produce C_2_ products during photocatalytic CO_2_ reduction (Figure 4b; Figure S26a). Although the product yield of individual sites Bi or S was higher than that of TiO_2_, the C_2_+ product was still not detected, which further proved the necessity of asymmetric double sites for C─C coupling. Control experiments further confirm that the generation of C_2_ products is significantly suppressed in the absence of any one of the three essential components, namely light, photocatalysts, or Na_2_SO_3_. (Figure S27). To confirm the carbon source of acetic acid, we performed a ^13^C isotope labelling experiment. ^13^C‐labeled NMR results clearly demonstrates that the carbon atoms in the produced acetic acid are exclusively derived from the photoreduction of CO_2_ (Figure S28), ruling out the possibility of carbon contamination from other potential sources (e.g., residual organic impurities in the reaction system). Notably, (Bi, S)TiO_2_ maintains excellent stability for CO_2_ photoreduction to CH_3_COOH even after prolonged operation, with the selectivity remaining at 89% (Figure 4c). This result underscores the promising potential of (Bi, S)TiO_2_ as an efficient and stable photocatalyst for converting CO_2_ high‐value products. The superior C_2_ production rate and selectivity of (Bi, S)TiO_2_ distinguish it from previously reported materials (Figure 4d and Table S6). Moreover, following long‐term CO_2_RR, the structural integrity and chemical states of (Bi, S)TiO_2_ remain unaltered (Figures S29–S32 and Table S7).

### 2.4 Mechanism of CO_2_RR

**FIGURE 5:**
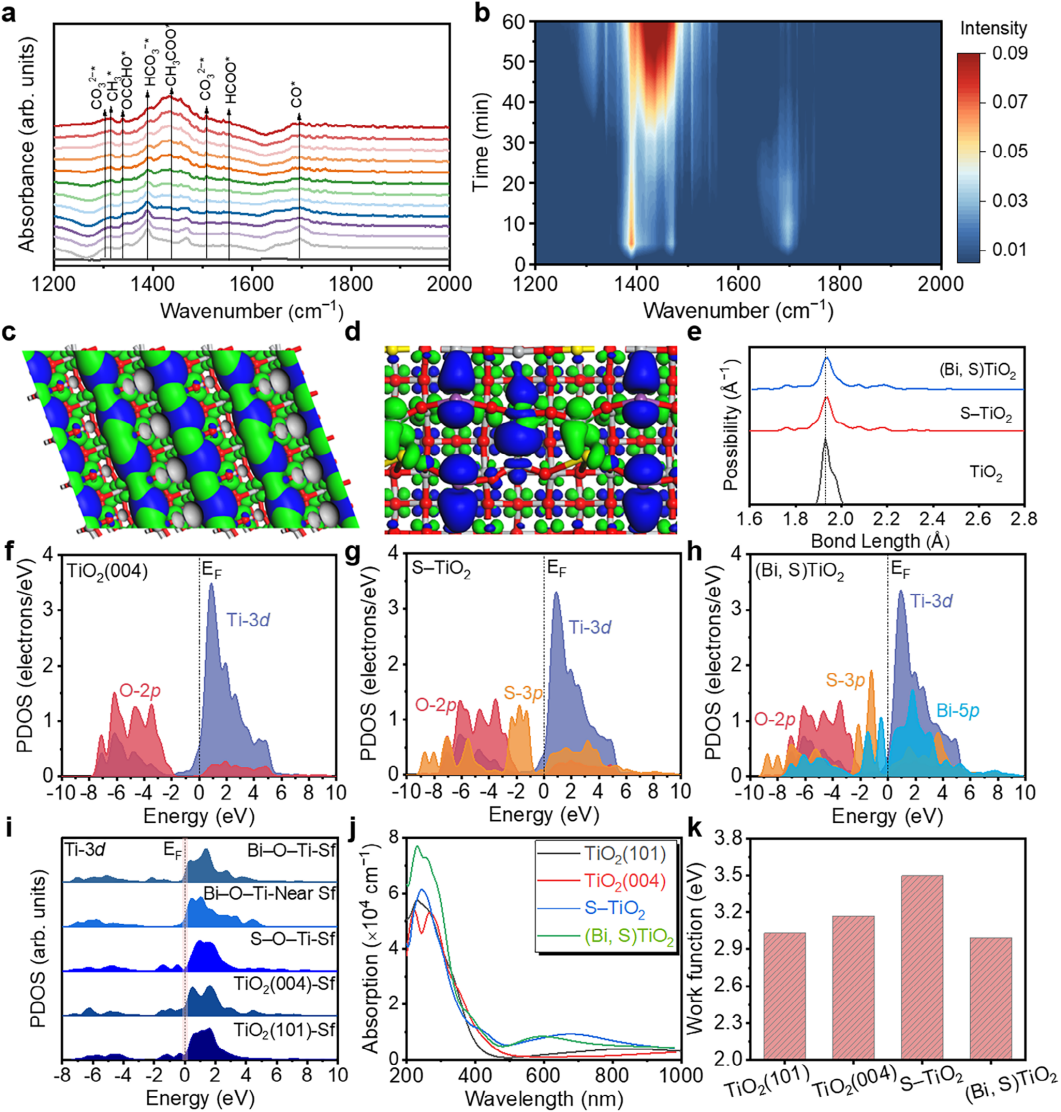
(a, b) The in‐situ FTIR spectra of (Bi, S)TiO_2_ for CO_2_RR. The electronic distributions of bonding and anti‐bonding orbitals near the Fermi level in (c) TiO_2_ (101) and (d) (Bi, S)TiO_2_. Blue isosurface = bonding orbitals and green isosurface = anti‐bonding orbitals. Purple balls = Bi, Yellow balls = S, Grey balls = Ti, and Red balls = O. (e) The bond length comparisons. The PDOSs of (f) TiO_2_(004), (g) S–TiO_2_, and (h) (Bi, S)TiO_2_. (i) The site‐dependent PDOS of Ti‐3*d*. (j) The comparisons of simulated absorption spectra. (k) The comparisons of work functions.

To elucidate the mechanism of the photocatalytic CO_2_ reduction, we used in‐situ Fourier transform infrared (FTIR) spectroscopy to track the reaction intermediates during the catalytic process [31, 32]. When monitoring the (Bi, S)TiO_2_ nanosheets under illumination for 60 min, new vibrational peaks were observed at intervals of 5 min (Figure 5a,b). These peaks’ intensities fluctuate with illumination time, associated with the dynamic balance of intermediate generation, conversion and consumption, originating from periodic changes in photogenerated charge carrier concentration and competitive adsorption on active sites. The peak at 1325 cm^−1^ (C─H bending vibration of hydrogenated intermediates, crucial for hydrocarbon products) increases gradually in the first 20 min, increases slightly between 20–40 min, and changes slowly afterward, possibly due to the balance of intermediate consumption and production. The peak at 1551 cm^−1^ (symmetric O═C─O vibration, assigned to COOH^*^ intermediates) shows a gradual trend that rapidly increases within 15 min and then stabilizes, indicating that COOH^*^ is progressively generated and converted until a stable equilibrium is reached, and it acts as a rate‐determining intermediate at a specific reaction stage [7]. Additionally, the peak at 1388 cm^−1^ arises from the symmetric stretching vibration of HCO_3_^−^, which forms due to strong CO_2_ adsorption at Lewis base site, a result of the interaction between CO_2_ and H_2_O (Figures S33 and S34) [33]. This peak stabilizes in the initial 5 min, then decreases with slight late‐stage fluctuation, reflecting equilibrium between CO_2_ adsorption‐HCO_3_^−^ formation and HCO_3_^−^ consumption as a precursor. Meanwhile, CO (1695 cm^−1^), as an important intermediate and one of the products from the conversion of COOH^*^, also demonstrated rapid generation and subsequent decrease due to the occurrence of CO─CO coupling. Subsequent coupling of two COOH intermediates may lead to the formation of CH_3_COOH via C─C coupling, evidenced by the peak at 1434 cm^−1^ (COO stretching vibration) [34]. This peak appears at 15 min, increases until 55 min, then fluctuates stably, indicating the product accumulation and balanced CH_3_COOH formation‐desorption. The peak at 1338 cm^−1^ (COCHO^*^, a typical CO^*^ coupling product and a crucial intermediate for CH_3_COOH) fluctuates synchronously with the 1434 cm^−1^ peak and appears slightly earlier than CH_3_COOH, confirming their sequential transformation in the reaction pathway [35].

To further analyse the surface charge distribution of (Bi, S)TiO_2_ and explore potential reaction pathways for CO_2_ to CH_3_COOH conversion, we studied the electronic structures and optical properties of (Bi, S)TiO_2_. For the pristine TiO_2_(101) surface, highly ordered electronic distributions of bonding and anti‐bonding orbitals were observed near the Fermi level (E_F_) at Ti sites (Figure 5c). After anchoring Bi single atoms on S–TiO_2_, it is noted that the electronic distributions of bonding orbitals are further enhanced, especially near the surface Bi SAs, indicating Bi SAs as the active sites to promote photocatalysis (Figure 5d). Surface S sites primarily contribute to anti‐bonding orbitals. For the TiO_2_ surface, the bond lengths are highly concentrated near 1.93 Å. The introduction of S has enlarged the bond lengths to 1.94 Å, while the further incorporation of Bi SAs has not significantly affected the bond lengths (Figure 5e). Detailed electronic structures of TiO_2_(004), S–TiO_2_, and (Bi, S)TiO_2_ were further investigated via projected density of states (PDOS) calculations based on density functional theory (details are in Experimental Section of Supporting Information). The PDOS analysis shows that pristine TiO_2_(004) displays that there is an evident gap between the valence band maximum (VBM) and the conduction band minimum (CBM), which are contributed by O‐2*p* and Ti‐3*d* orbitals, respectively (Figure 5f). With the introduction of S doping, it is noted that S‐3*p* orbitals are mainly located between the O‐2*p* and Ti‐3*d* as the additional electron transport path, allowing electrons to cross the energy gap with lower energy costs to facilitate the electron transfer efficiency in the materials (Figure 5g) [36]. However, the limited overlap between S‐3*p* and O‐2*p* orbitals likely contributes to the observed lattice distortion. Additionally, the narrowed bandgap enhances visible‐light absorption, which is beneficial for driving photocatalysis. After introducing Bi SAs into the (Bi, S)TiO_2_, Bi‐5*p* orbitals introduce abundant states within the bandgap, which act as temporary storage for photogenerated charges, preventing the fast recombination and ensuring more carriers are available for CO_2_ conversion during photocatalysis (Figure 5h). Although Bi‐5*p* orbitals dominate the VBM, there is a bandgap to enable the absorption of light and initiate the electrons‐holes separation process. Compared with pristine TiO_2_(004), the incorporation of Bi and S induces upshifting and downshifting of O‐2*p* and Ti‐3*d* orbitals with enhanced overlap with S‐3*p* orbitals, which guarantees the efficient electron transfer during the photocatalysis. Site‐dependent PDOS of Ti‐3*d* orbitals also reveals the electronic modulations by exposed surface, S doping, and Bi SAs. Compared to the TiO_2_(101) and (004) surfaces, the introduction of only S will not improve the electron density near E_F_ (Figure 5i). With the incorporation of Bi SAs, the Ti‐3*d* orbitals downshift with a significant increase in electron density near the Fermi level, which enhances the concentration of active electrons and improves the surface activity of TiO_2_. The optical properties also confirm the critical contributions of S doping and Bi SAs (Figure 5j). Compared to pristine TiO_2_ surfaces, the introduction of S doping and Bi SAs substantially increases light absorption intensity in the visible range, which enhances photocatalytic performance. In addition, the work function can be considered as the energy threshold for electrons to escape from the catalyst. The work function comparisons among different samples exhibit an order of (Bi, S)TiO_2_< TiO_2_(101) < TiO_2_(004) < S–TiO_2_ (Figure 5k). Even though the S introduction has enlarged the work function, the Bi SAs have obviously reduced the work function of the catalyst. Due to the lowering of the electron escape threshold on the (Bi, S)TiO_2_, it becomes easier for photoexcited electrons to participate in CO_2_ reduction. More importantly, the reduced work function also makes the catalyst surface more favourable for the adsorption of target intermediates.

**FIGURE 6:**
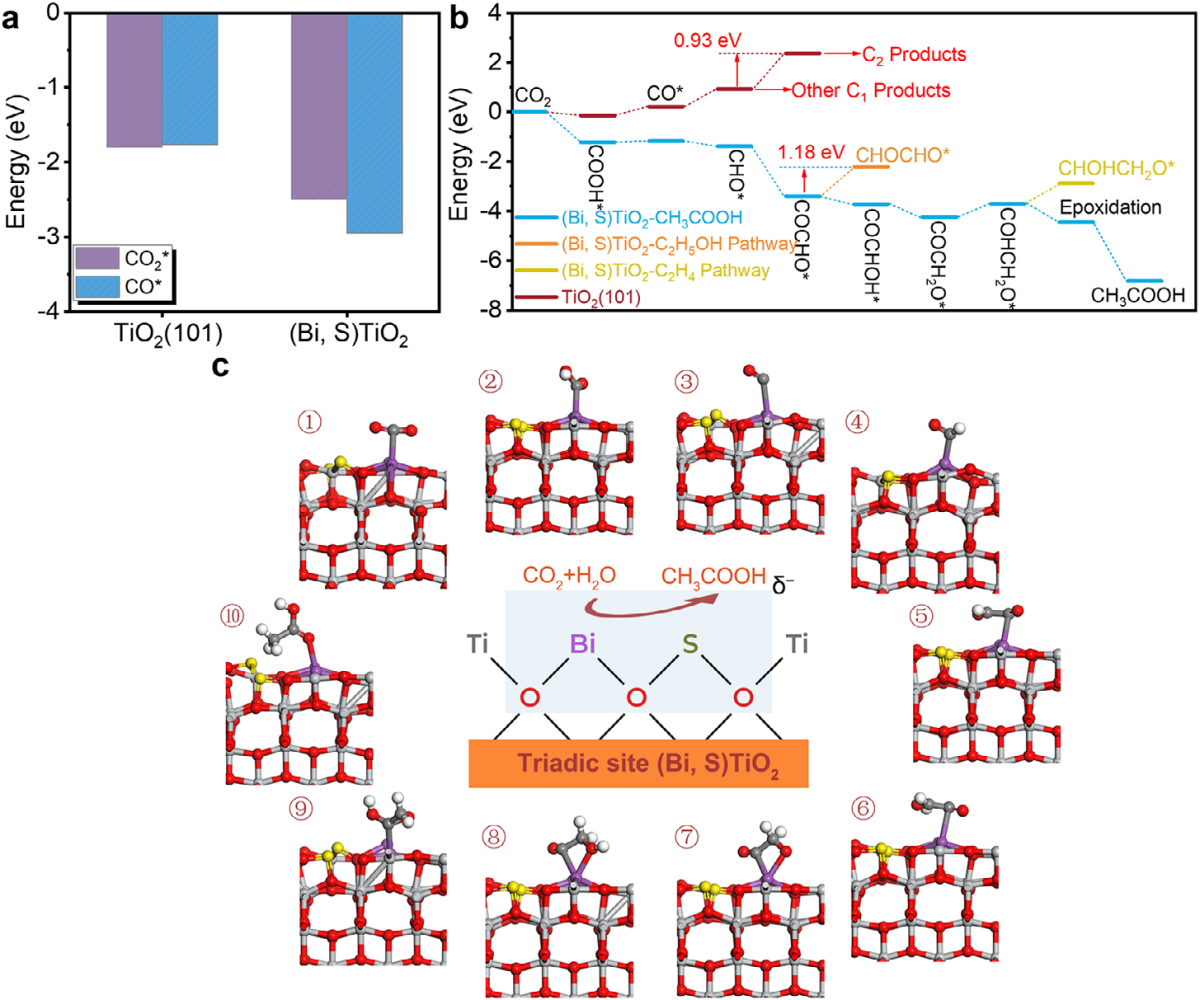
(a) The adsorption energies of CO_2_^*^ and CO^*^ in TiO_2_(101) and (Bi, S)TiO_2_. (b) The reaction energy trends for CO_2_RR toward different products. (c) The triadic Bi and S single atoms containing (SO_2–_BiO_4_)^δ−^ structure and photocatalytic CO_2_ reduction pathway.

We further explored the adsorption preferences of key intermediates (CO_2_^*^ and CO^*^) via DFT simulations, which determine the initial activation of reactants and C─C coupling (Figure 6a). Compared with pristine TiO_2_(101), (Bi, S)TiO_2_ shows much stronger adsorption preference for both CO_2_^*^ and CO^*^, leading to faster CO_2_ activation and more favourable C–C coupling. This enhanced adsorption is primarily attributed to Lewis base sites structure of (SO_2–_BiO_4_)^δ−^. The overall reaction energy profiles of TiO_2_(101) and (Bi, S)TiO_2_ are shown in Figure 6b,c. For pristine TiO_2_(101), it is noted that an energy‐consuming step is required for the further reduction of CO^*^ due to large barriers to proceed. In particular, the formation of CHO^*^ encounters a barrier of 0.70 eV, which limits the production of C1 products. The C─C coupling exhibits an even higher barrier of 0.93 eV, largely suppressing the formation of C_2_ products via photocatalysis. In contrast, (Bi, S)TiO_2_ shows energetically favourable trends for the generation of CHO^*^ and C─C coupling, resulting in a high selectivity toward C_2_ products. For the hydrogenation of COCHO^*^, the reaction pathway toward specific products is determined by the reaction site, where the formation of COCHOH^*^ is much more favourable than that of CHOCHO^*^ (which encounters a large barrier of 1.18 eV). This distinct preference explains the high selectivity for CH_3_COOH. The hydrogenation of COCH_2_O^*^ is the rate‐determining step with an energy barrier of 0.53 eV. The subsequent step determines the competition between CH_3_COOH and C_2_H_4_, where a stronger tendency for epoxidation favours higher selectivity for CH_3_COOH over C_2_H_4_. Therefore, the optimized electronic structures of (Bi, S)TiO_2_ are the key factor underlying its significantly improved photocatalytic CO_2_RR performances. The reasoning for the high selectivity from the electronic structure's point of view needs to be expanded.

## 3 Conclusion

In summary, we have developed a triadic‐structural photocatalyst for the selective photoreduction of CO_2_ to the C_2_ product acetic acid, achieved by coupling single‐atom Bi species with a 3D porous single‐crystal S‐doped TiO_2_. The 3D porous architecture of the catalyst facilitates efficient mass transfer during the photocatalytic reaction, addressing a key limitation of traditional single‐crystal materials. The introduction of single‐atom Bi and S creates asymmetric active sites within the TiO_2_ substrate and induces intermediate energy bands in the bandgap, resulting in exceptional catalytic efficiency for CO_2_ conversion. Specifically, the S–O_2_ coordination structure promotes the formation of Lewis base sites ((SO_2–_BiO_4_)^δ−^), ensuring strong adsorption of CO_2_ and subsequent C─C coupling while lowering the energy barriers of rate‐limiting steps in photocatalysis with a high selectivity toward the C_2_ product. The specifically designed photocatalytic system achieves a high acetic acid conversion rate of 66.7 µmol g^−1^ h^−1^ with over 89% selectivity. This research paves the way for the development of high‐efficiency photocatalysts by integrating single‐atom active sites with porous single‐crystal materials for advancing CO_2_ utilization technologies.

## Funding

Research Grants Council (RGC) of Hong Kong (Projects 14304019, 17300424, 15304023, and 15304724), the RGC‐TRS (T23‐713/22‐R), the HK Environment and Conservation Fund (ECF 2021–152; ECF2021‐141), the NSFC/RGC Joint Research Scheme (N_PolyU502/21), NSFC/RGC Collaborative Research Scheme (CRS_PolyU504/22), the European Union‐Hong Kong Research Cooperation Co‐funding Mechanism by the Research Grants Council sponsored by the RGC (E‐HKU701/23 and GH2‐101070721), the National Youth Talent Program (006170130648), the Opening Project of State Key Laboratory of High Performance Ceramics (SKL202502SIC), the Heilongjiang Provincial Natural Science Foundation of China (YQ2025B004), and the Hainan Provincial Natural Science Foundation of China.

## Conflicts of Interest

The authors declare no conflict of interest.

## Supporting information




**Supporting File**: adma72600‐sup‐0001‐SuppMat.pdf.

## Data Availability

All the data supporting the findings of this study are available from the corresponding author upon reasonable request.
