# Supplementary material for: Asymmetric Bi and S Single Atoms Over Porous Single‐Crystal TiO2 for Efficient CO2 Photoreduction to Acetic Acid
Source: Adv Mater. 2026 Feb 18;38(16):e17586. doi: 10.1002/adma.202517586 (PMC12994305; doi:10.1002/adma.202517586)
Supplement: Supplementary file 1 — Supporting File: adma72600‐sup‐0001‐SuppMat.pdf. [file ADMA-38-e17586-s001.pdf]

Supporting Information

Asymmetric Bi and S Single Atoms over Porous Single-Crystal TiO_2 for Efficient CO_2 Photoreduction to Acetic Acid

Guangri Jia, Ying Wang, Mingzi Sun, Yingchuan Zhang, Zhipeng Xie, Xiaoqiang Cui, Bolong Huang, Jimmy C. Yu, and Zhengxiao Guo**

E-mail: zxguo@hku.hk; b.h@cityu.edu.hk

Table of Contents

Experimental Section 4

Figure S1. XRD pattern of pristine single-crystal $Bi_4Ti_3O_{12}$. 7

Figure S2. Raman spectra of pristine single-crystal $Bi_4Ti_3O_{12}$. 8

Figure S3. TEM image of pristine single-crystal $Bi_4Ti_3O_{12}$. 9

Figure S4. SEM images of $Bi_4Ti_3O_{12}$ treated without and with sulphuration. 10

Figure S5. High-resolution XPS spectra of pristine single-crystal $Bi_4Ti_3O_{12}$. 11

Figure S6. Powder XRD pattern analysis of sulfurized single-crystal $Bi_4Ti_3O_{12}$. 12

Figure S7. TEM image and corresponding mapping for sulfurized single-crystal $Bi_4Ti_3O_{12}$. 13

Figure S8. High-resolution XPS spectra for sulfurized single-crystal $Bi_4Ti_3O_{12}$. 14

Figure S9. High-resolution XPS spectra of S 2*p*. 15

Figure S10. SEM images of (Bi, S)TiO_2. 16

Figure S11. TEM images of (Bi, S)TiO_2. 17

Figure S12. TEM images and SAED pattern of TiO_2. 18

Figure S13. Raman spectra of TiO_2 and (Bi, S)TiO_2. 19

Figure S14. Specific surface area analysis of TiO_2 and (Bi, S)TiO_2. 20

Figure S15. High-resolution XPS spectra of O 1*s* and Ti 2*p* for (Bi, S)TiO_2. 21

Figure S16. Structure analysis based on S K-edge X-ray absorption spectra. 22

Figure S17. EXAFS experimental results and theoretical fits of (Bi, S)TiO_2. 23

Figure S18. Wavelet transform analysis of the k^2-weighted EXAFS data of Ti K-edge. 24

Figure S19. Wavelet transform analysis of the k^2-weighted EXAFS data of S K-edge. 25

Figure S20. *k* space fitting curve of the Ti K-edge, Bi L-edge, and S K-edge. 26

Figure S21. UV-visible light absorption spectra. 27

Figure S22. UPS spectra analysis. 28

Figure S23. PL spectra analysis. 29

Figure S24. EIS and photocurrent curves analysis. 30

Figure S25. NMR analysis of photocatalytic CO_2 reduction. 31

Figure S26. Products analysis of the time-dependent photocatalytic CO_2RR for TiO_2. 32

Figure S27. Control experiment of photocatalytic CO_2 reduction reaction. 33

Figure S28. The photocatalytic ^{13}C-labeled CO_2 reduction to acetic acid of (Bi, S)TiO_2. 34

Figure S29. The post-reaction XRD pattern of (Bi, S)TiO_2 after long-term CO_2RR. 35

Figure S30. High-resolution XPS spectra for (Bi, S)TiO_2 after long-term CO_2RR. 36

Figure S31.The SEM images of (Bi, S)TiO_2 after long-term CO_2RR. 37

Figure S32. Specific surface area analysis of (Bi, S)TiO_2 after long-term CO_2RR. 38

Figure S33. CO_2-TPD signal analysis. 39

Figure S34. TPD-Mass signal analysis. 40

Table S1. The element content of (Bi, S)TiO_2 and TiO_2 from ICP-OES. 41

Table S2. EXAFS curves fitting parameters of Bi L-edge and S K-edge. 42

Table S3. The energy band of (Bi, S)TiO_2 and TiO_2. 43
Table S4. TA spectra carriers' kinetics and fitting parameters. 44
Table S5. The parameter of TRPL of (Bi, S)TiO_2 and TiO_2. 45
Table S6. Comparison of CO_2RR to C_2 products with those of reported photocatalysts. 46
Table S7. The element content of (Bi, S)TiO_2 after CO_2RR reaction from ICP-OES. 47
Supporting References 48

Experimental Section

Chemicals: Bismuth oxide (Bi_2O_3, 99.9%), sodium sulfite (Na_2SO_3, 99.99%), titanium dioxide (TiO_2, anatase, 99.8%), sodium sulfate anhydrous (Na_2SO_4, 99.99%), sodium chloride (NaCl, 99.8%), $Bi(NO_3)_3 \cdot 5H_2O$ (99%), $Na_2S \cdot 9H_2O$ (98%), and potassium chloride (KCl, 99.8%) were purchased from Aladdin Chemical Co., Ltd. CO_2 was used for CO_2 reduction reaction with a purity of 99.995% (Linde.hk). Dimethylsulfoxide (DMSO, 99.9%) was obtained from Sigma-Aldrich, USA. The reagents used in the experiment were of analytical purity and were used without further purification.

Synthesis of pristine BTO single-crystal nanosheets: The pristine BTO nanosheets were synthesized using a conventional solid-state reaction methodology. A precise combination of Bi_2O_3, TiO_2, KCl, and NaCl with a calculated mole ratio of Bi:Ti:K:Na at 4:3:160:160 underwent thorough grinding for 15 minutes in a mortar. Subsequently, the mixture was annealed at a high temperature of 800 °C for 12 hours in a controlled static air, with a gradual ramping rate of 5 °C min^{-1}. Following the annealing process, the sample was washed using deionized water and ethanol to eliminate any residual KCl and NaCl for three times. Ultimately, the resultant powder was dried at 60 °C overnight and then stored for subsequent use.

Synthesis of single-crystal porous (Bi, S)TiO_2 nanosheets: 200 mg of BTO was dispersed and sonicated in a 35 mL water as solution A. Simultaneously, 300 mg of thioacetamide was dissolved in 5 mL of water as solution B. Subsequently, solution B was added to solution A while maintaining a constant temperature of 60 ºC with continuous stirring for 30 minutes. The suspension was accompanied by a change in color from white to yellowish–brown. The yellowish–brown solution was encased in a Teflon-lined stainless-steel autoclave at 180 ºC for 16 hours. Subsequently, the obtained powder was dispersed in a 4 M HNO_3 solution and stirred for 30 minutes to eliminate Bi_2S_3, thereby ensuring that the powder surface retains residual S and Bi species. The obtained powder was then centrifuged and washed with water and ethanol for three times. The powder was further annealed at 450 °C in air for 2 hours to obtain the final (Bi, S)TiO_2 sample. For Bi-TiO_2 and S-TiO_2 samples, the commercial anatase TiO_2 was selected as support to load Bi single atoms and S single atoms, aiming to explore the influence of different single-atom sites on the catalytic CO_2 reduction activity. An amount of Bi^{3+} (from $Bi(NO_3)_3$) or S^{2-} (from Na_2S) was pre-adsorbed on the surface TiO_2 powder after stirring overnight. The obtained powder then centrifuged and washed with water and ethanol for three times. The powder was further annealed at 450 °C in air for 2 hours to obtain the final samples.

Characterizations: Powder XRD was recorded on an X-ray diffractometer from Bruker, Germany using a Cu-Kα source (0.15418 nm). SEM images were collected using LEO 1530. TEM images were obtained using a Thermo Scientific Talos F200X system. XPS was obtained using a ThermoFisher (ESCALAB Xi^+ USA). A micro-Raman spectrometer was conducted to obtain

Raman spectra using a 532 nm laser as excitation source. UV-vis absorption spectra were performed using UV–VIS–NIR spectrophotometer (Agilent Cary 5000). PL and time-resolved TRPL spectra were obtained using Edinburgh FLS1000 spectrophotometer with excitation wavelengths of 375 nm. TA spectroscopy was conducted using an optical setup consisting of a frequency-doubled mode-locked Ti:sapphire femtosecond laser (a pulse width of 35 fs and a repetition rate of 1 kHz) from coherent and an optical parametric amplifier (OPA) system. The seed pulses were split into two beams: one beam was directed to the OPA system to generate a 400 nm pump laser pulse, while the other beam was focused on a sapphire crystal to produce a white light continuum spanning 450–700 nm for UV–vis probe light. FTIR spectra were obtained using Nicolet iS50 (ThermoFisher). BET analysis was done from Micromeritics ASAP 2460. XAFS investigation was carried out at the BL08B2 of SPring-8 in Japan, utilizing X-ray beam with 8 GeV and 100 mA. The X-ray beam was monochromatized using a water-cooled Si (111) double-crystal monochromator and focused with two Rh-coated focusing mirrors, resulting in a beam size of 2.0 mm horizontally and 0.5 mm vertically at the sample position. ICP-OES was performed at iCAP 7600 (Thermo Fisher, Inc, USA).

Photocatalytic CO_2RR reaction: Photocatalytic reactions were conducted in an overhead-irradiation-type reactor featuring a closed gas circulation system (Labsolar-6A, Beijing Perfectlight, China). An appropriate amount of photocatalyst was dispersed in 30 mL of 0.1M Na_2SO_3 aqueous solution. Before exposing the reactor to a 300 W xenon lamp (300 mW/cm^2, PLS-SXE300, Beijing Perfectlight, China), the reactor was subjected to a degassing process under vacuum conditions, followed by purging with high-purity CO_2 for 30 minutes, and the internal pressure of the reactor was subsequently maintained at 1 atm. The reaction setup was maintained at 288 K with the aid of a cooling water system. To confirm CO_2 as the carbon source, control tests and $^{13}CO_2$ isotopic experiments were conducted. For $^{13}CO_2$ experiments, 99% ^{13}C-labeled CO_2 was used. The resulting gas products were analyzed using gas chromatography (Shimadzu 2030) with argon as the carrier gas, equipped with both a thermal conductivity detector and a flame ionization detector. The liquid products were analyzed using NMR.

The corresponding apparent quantum yields (AQY) for CO_2 reduction to acetate were quantitatively determined using the following equation:

$$\text{AQY}(\%) = \frac{\mathbf{8 \times N \times N_A \times h \times c}}{\mathbf{P \times S \times \lambda}} \times 100\%$$

wherein, N_A denotes the Avogadro constant (6.022×10^{23} mol^{-1}), N represents the acetate production rate, P is the specific light intensity of the target wavelength (mW·cm^{-2}), λ is the specific light wavelength (nm), h stands for the Planck constant (6.626×10^{-34} J·s), c is the speed of light in vacuum (3.00×10^8 m·s^{-1}), and S refers to the illumination area. Based on this equation, the AQY values were calculated as 0.124% at 350 nm and 0.083% at 380 nm, respectively.

Photoelectrochemical measurement: Photocurrent and EIS analyses were performed utilizing a

CHI650D electrochemical workstation in the presence of a 0.5 M Na_2SO_4 electrolyte under 300 W Xe lamp illumination. The setup employed a three-electrode system consisting of a platinum foil counter electrode, an Ag/AgCl reference electrode, and FTO-coated samples as the working electrode. EIS measurements were taken across a frequency spectrum ranging from 10^5 to 10^{-1} Hz with a 10 mV of amplitude.

DFT details: The theoretical calculations in this work were performed based on the DFT embedded in the CASTEP packages to explore the electronic modulations of photocatalysis in (Bi, S)TiO_2.[1] The exchange-correlation interactions were described within the framework of generalized gradient approximation with Perdew–Burke–Ernzerhof functionals.[2-4] A cutoff energy of 380 eV was adopted with the ultrasoft pseudopotentials, corresponding to an ultrafine quality setting. For structural energy minimization, the k-point grid was set to coarse quality, and the Broyden–Fletcher–Goldfarb–Shannon algorithm was employed.[5]

For the pristine TiO_2, the (101) and (004) surfaces with four-layer thicknesses were cleaved in 3 × 3 × 1 supercells. For S–TiO_2 and (Bi, S)TiO_2, 5% S and 5% Bi dopants were introduced in the Ti sites on the surface. For (Bi, S)TiO_2, 20 Å vacuum space was introduced along the z-axis to provide sufficient space for the adsorptions of different intermediates. The following convergence criteria were applied for geometry optimizations, where the Hellmann–Feynman forces should not exceed 0.001 eV/Å, and the total energy difference should not be over 5×10^{-5} eV/atom.

Figures

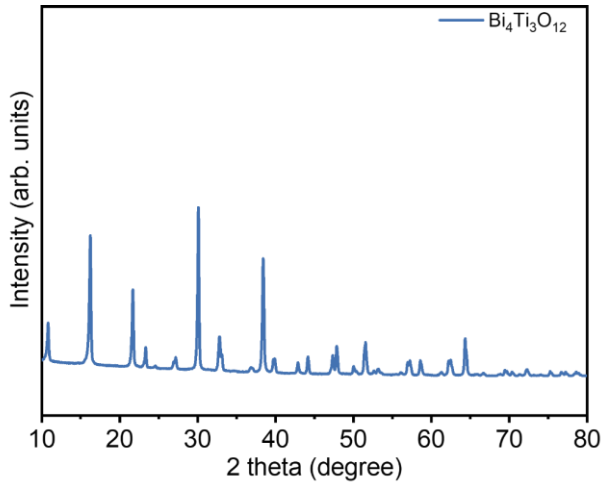

Figure S1. XRD pattern of pristine single-crystal $Bi_4Ti_3O_{12}$.

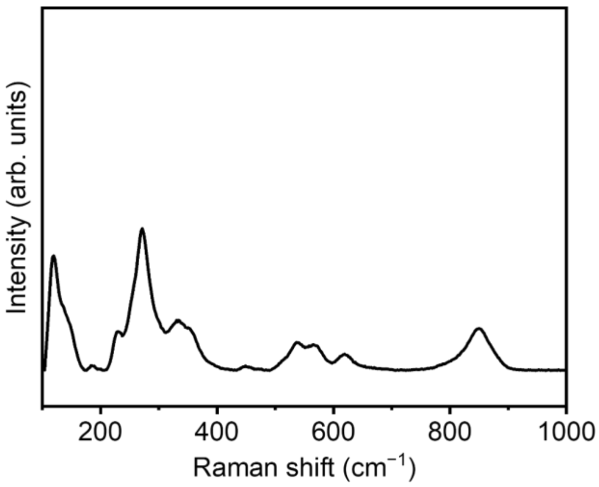

Figure S2. Raman spectra of pristine single-crystal $Bi_4Ti_3O_{12}$.

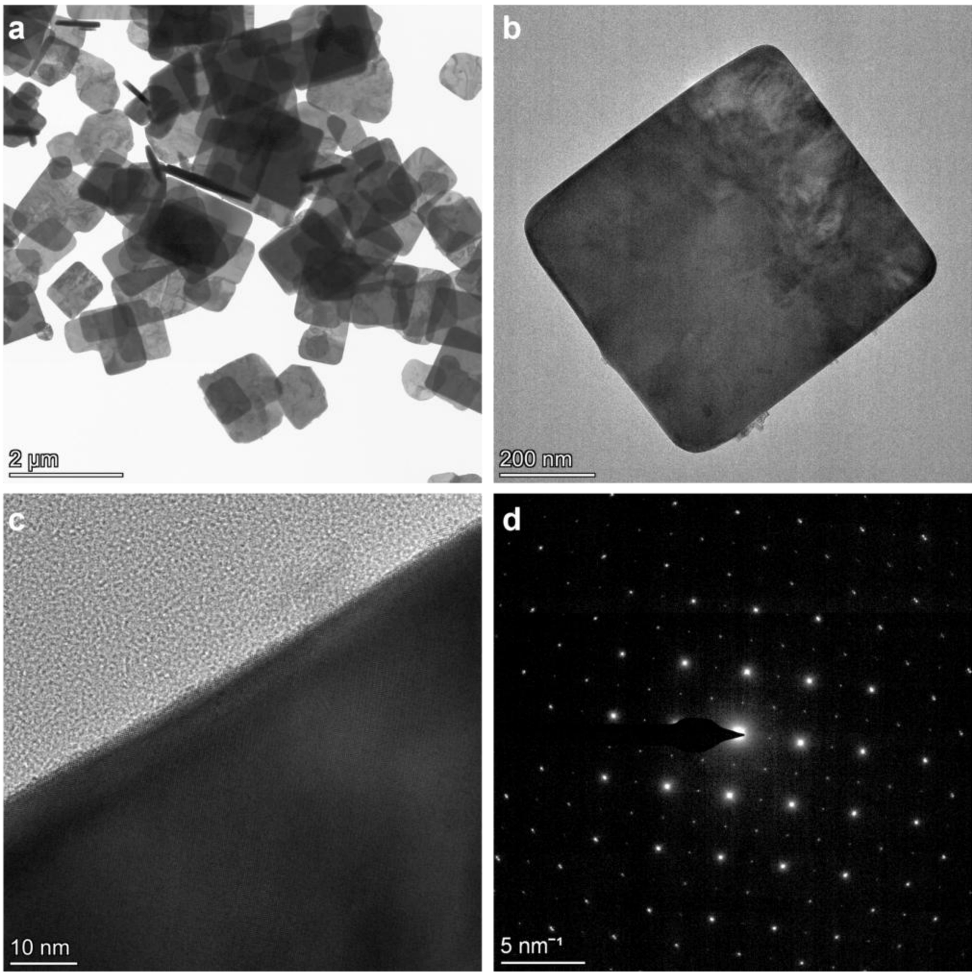

Figure S3. (a–b) TEM images, (c) High-resolution TEM image, and (d) corresponding SAED pattern of pristine single-crystal $Bi_4Ti_3O_{12}$.

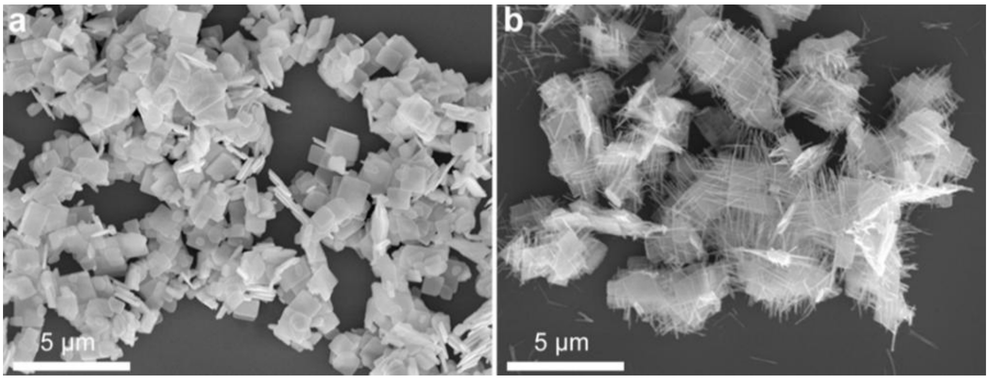

Figure S4. SEM images of $Bi_4Ti_3O_{12}$ treated without (a) and with (b) sulphuration.

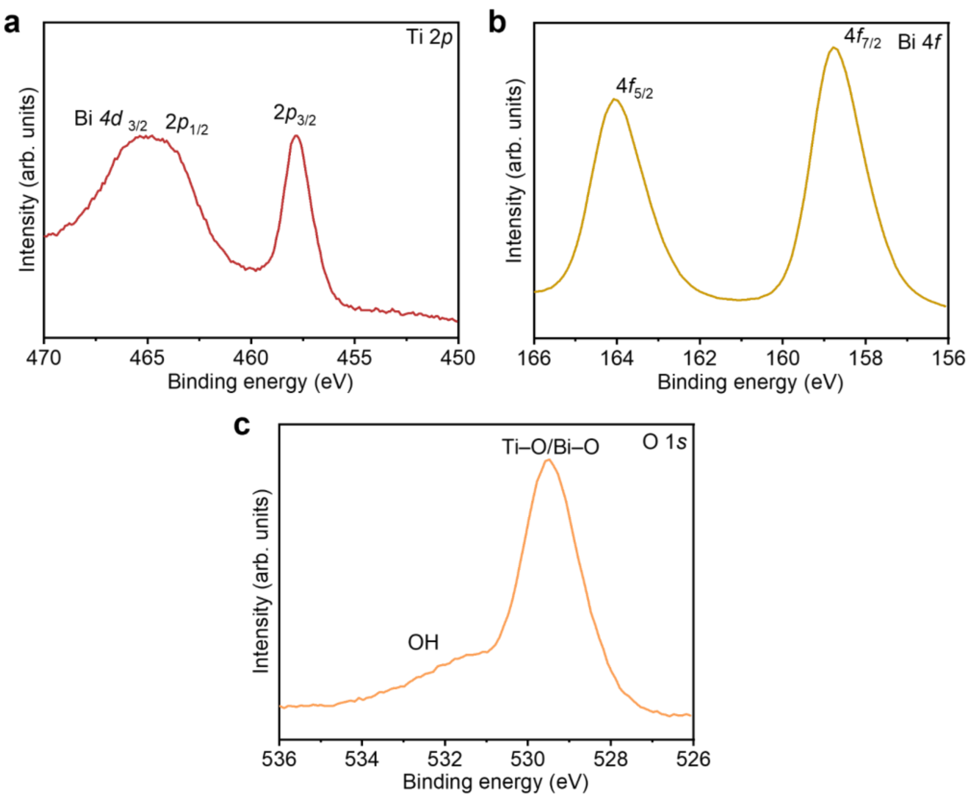

Figure S5. High-resolution XPS spectra of (a) Ti *2p*, (b) Bi *4f*, and (c) O *1s* of pristine single-crystal $Bi_4Ti_3O_{12}$.

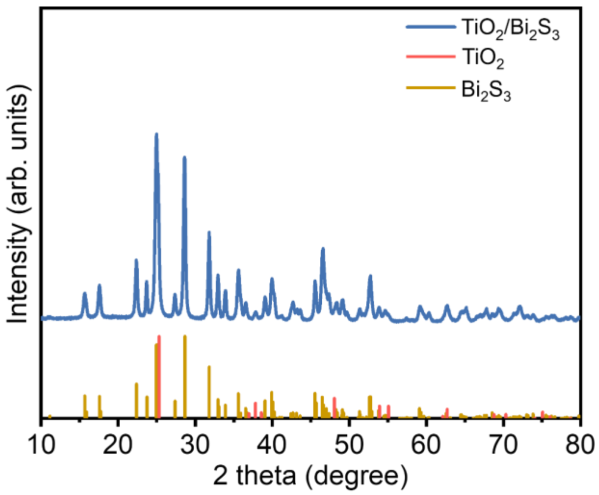

Figure S6. Powder XRD pattern analysis of sulfurized single-crystal $Bi_4Ti_3O_{12}$, standard Bi_2S_3 (PDF#84-0279), and TiO_2 (PDF#84-1285).

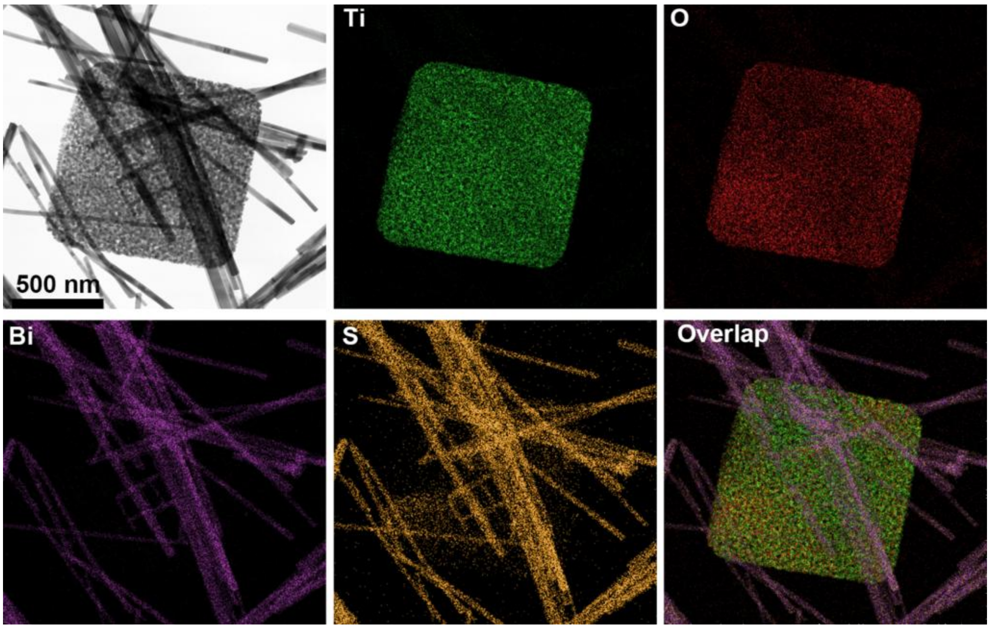

Figure S7. TEM image and corresponding mapping for sulfurized single-crystal $Bi_4Ti_3O_{12}$.

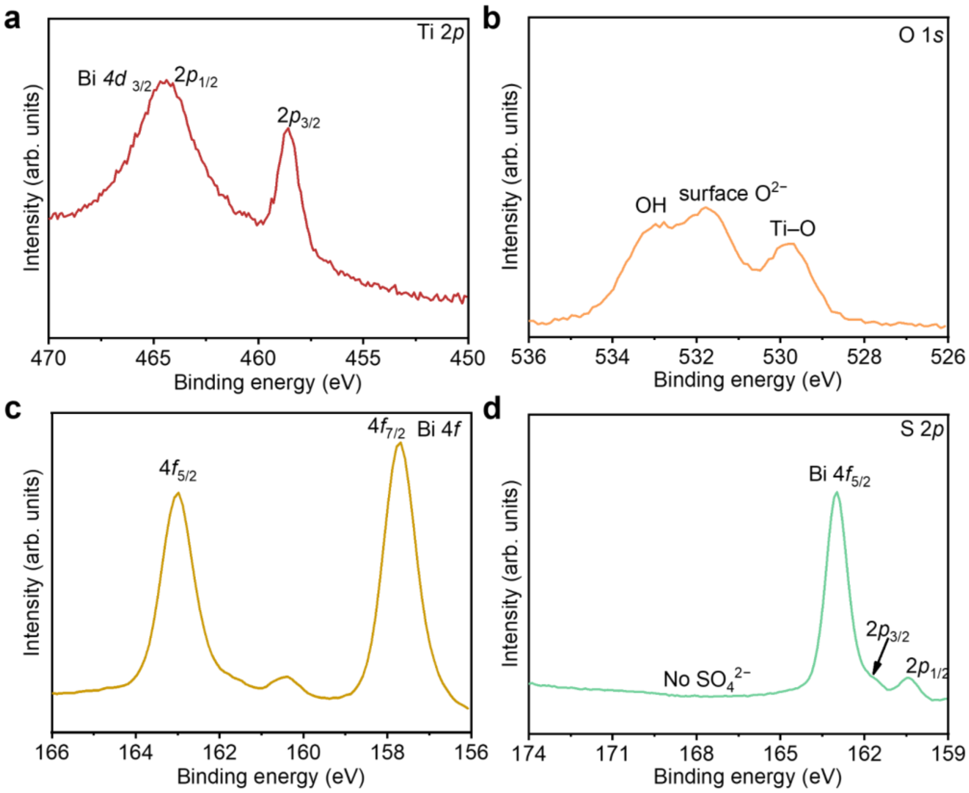

Figure S8. High-resolution XPS spectra of (a) Ti 2*p*, (b) O 1*s*, (c) Bi 4*f*, and (d) S 2*p* for sulfurized single-crystal $Bi_4Ti_3O_{12}$.

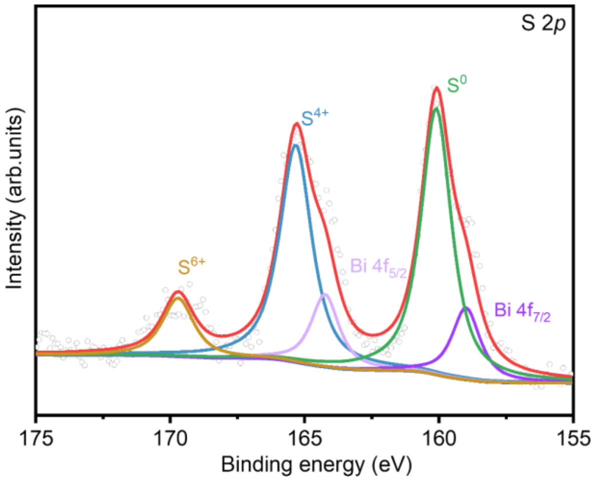

Figure S9. High-resolution XPS spectra of S 2*p* before annealing and after sulfidation and acid treatment.

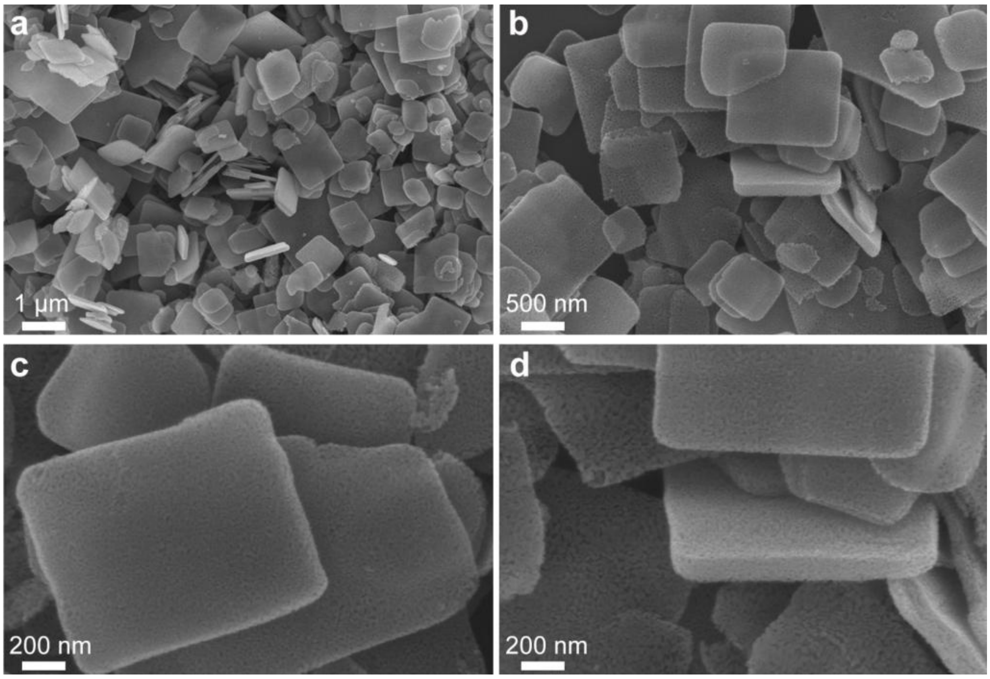

Figure S10. SEM images of (Bi, S)TiO_2.

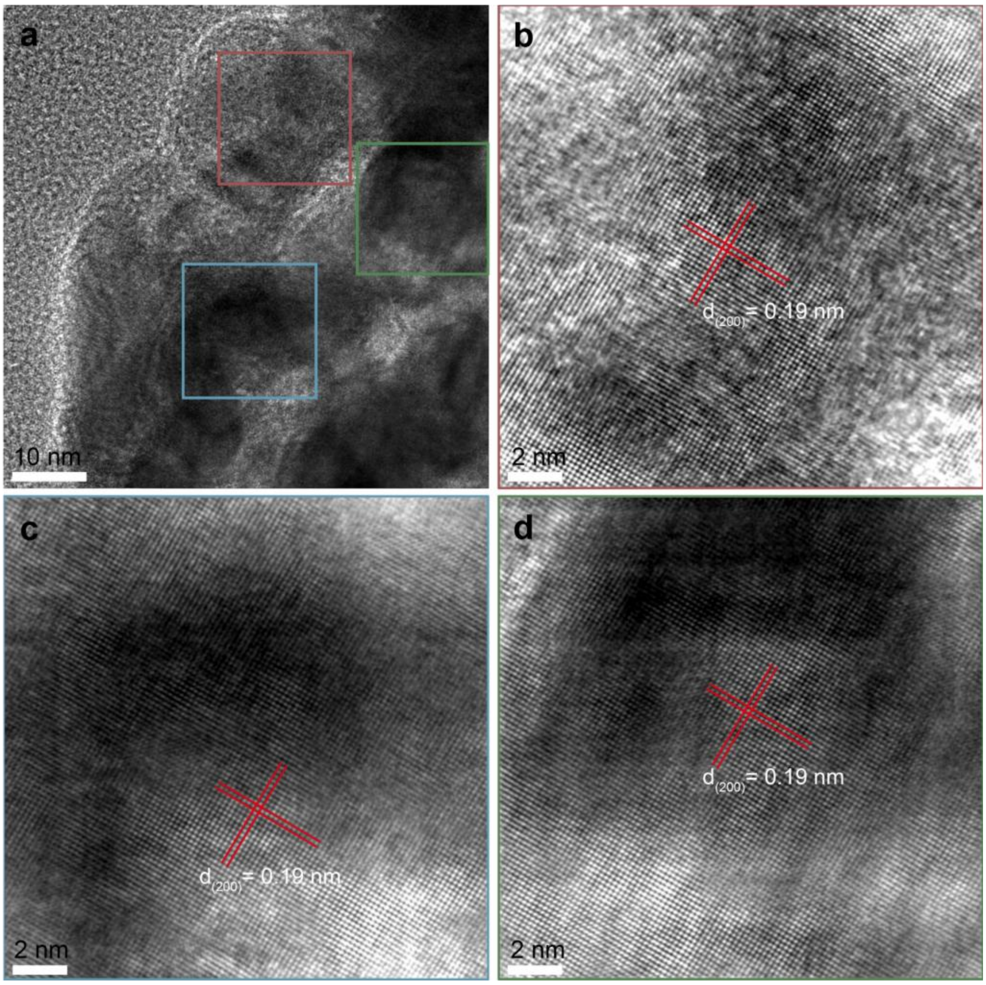

Figure S11. (a) High-resolution TEM image and (b–d) corresponding enlarged images in (a) of (Bi, S)TiO_2.

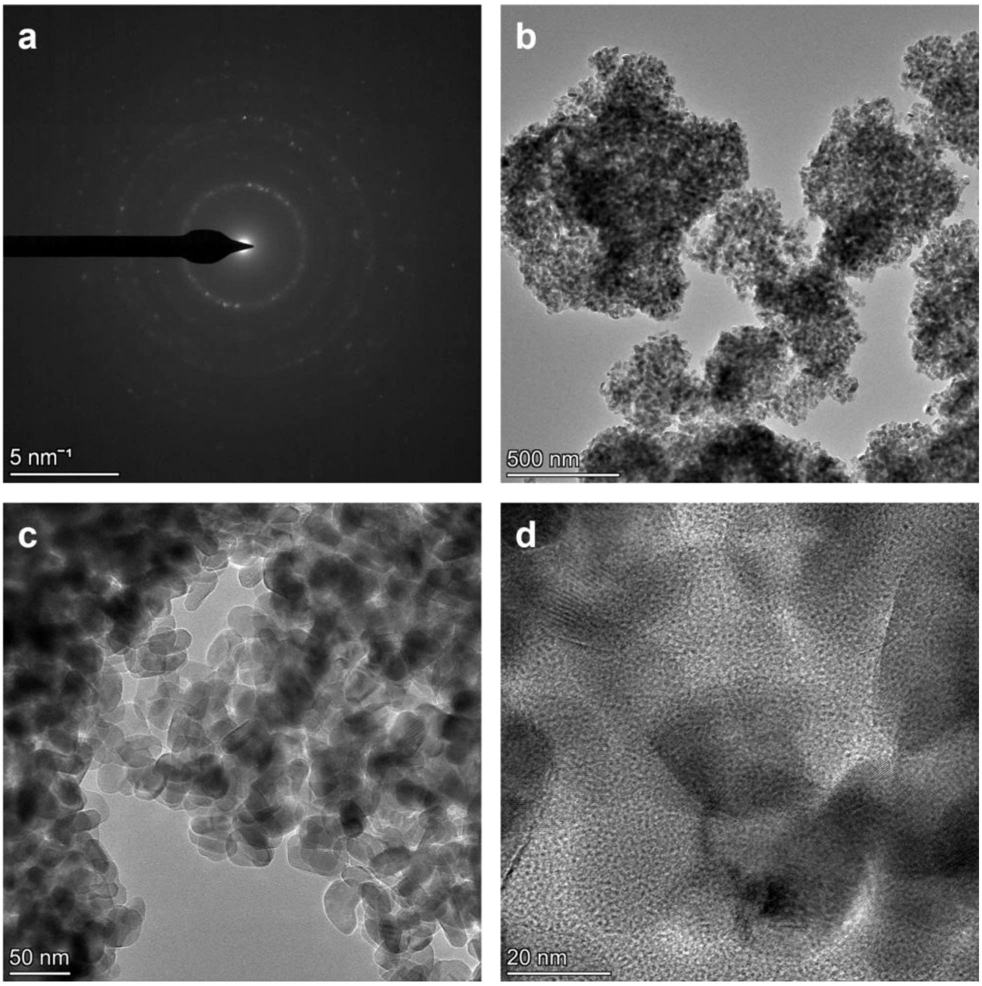

Figure S12. (a) SAED pattern, (b–c) TEM images, and (d) High-resolution TEM image of TiO_2.

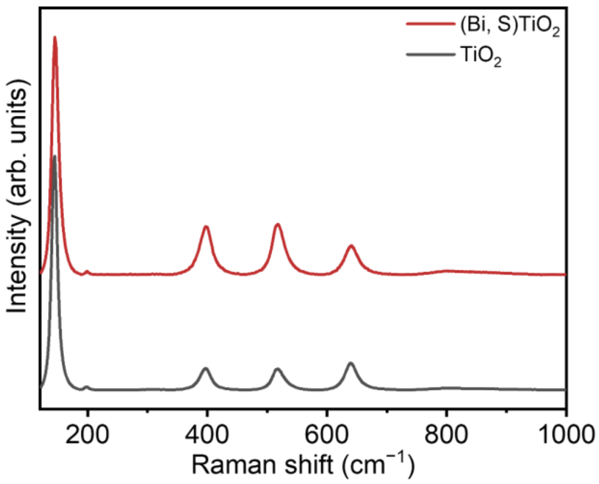

Figure S13. Raman spectra of TiO_2 and (Bi, S)TiO_2 with 532 nm laser excitation.

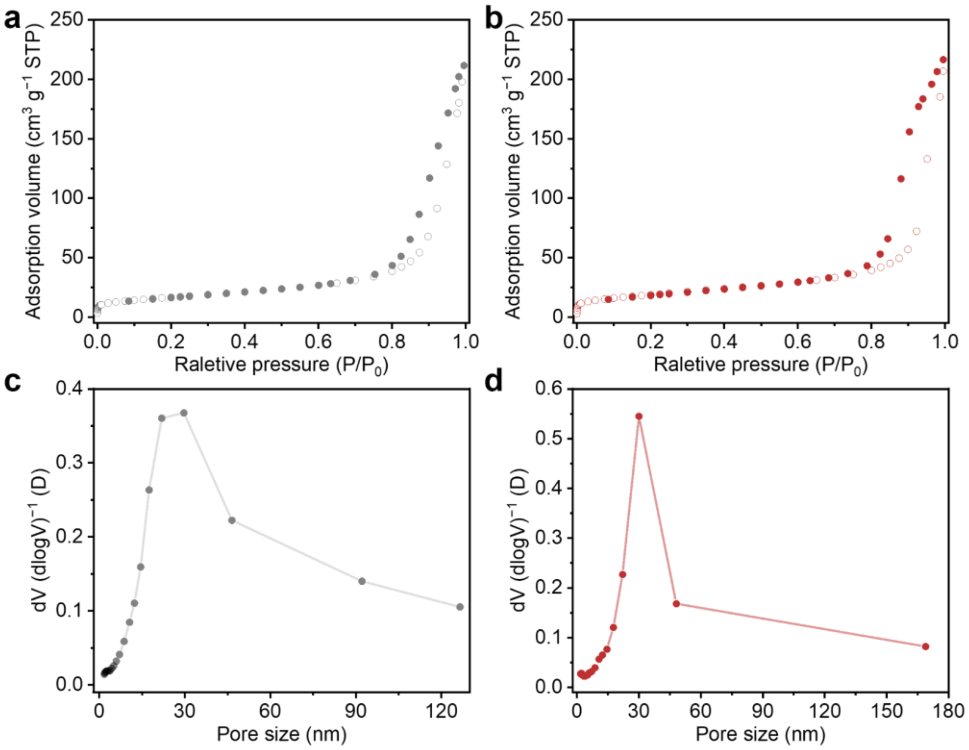

Figure S14. Adsorption–desorption isotherms and pore size distribution of (a, c) TiO_2 and (b, d) (Bi, S)TiO_2 with specific surface area of 66.3 m^2 g^{-1}.

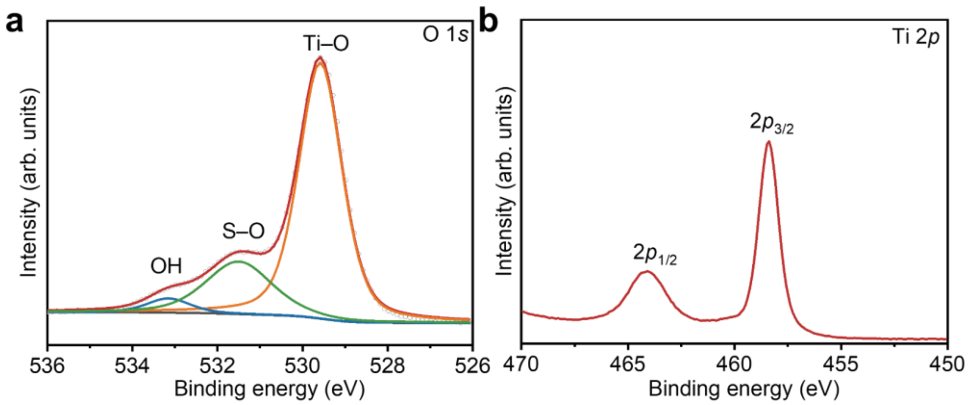

Figure S15. High-resolution XPS spectra of (a) O 1*s* and (b) Ti 2*p* for (Bi, S)TiO_2.

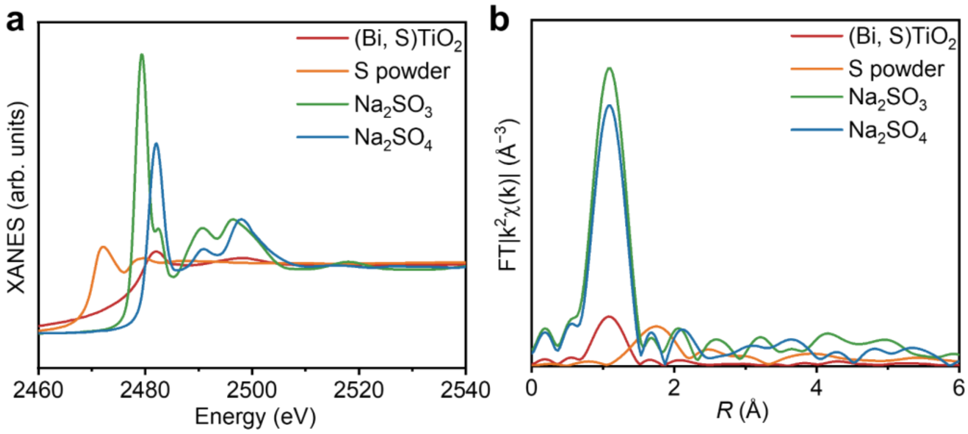

Figure S16. (a) S K-edge XANES spectra and (b) EXAFS spectra of (Bi, S)TiO_2, Na_2SO_3, Na_2SO_4, and S powder.

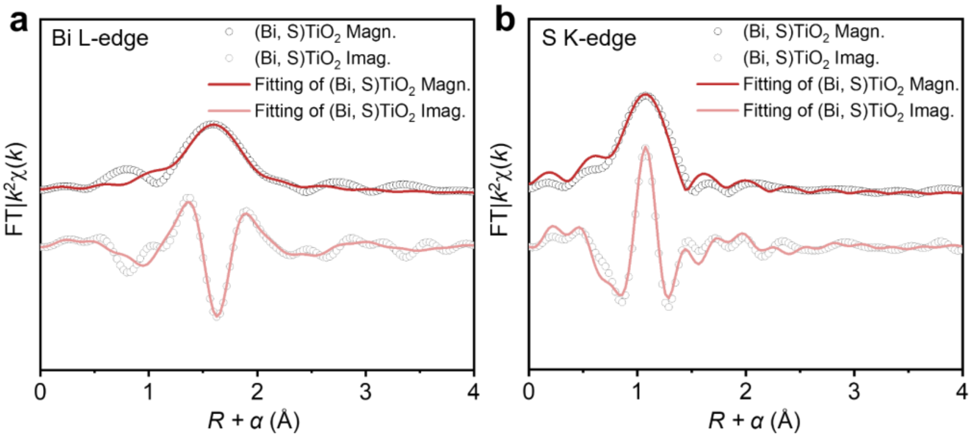

Figure S17. (a) Bi L-edge k^2-weighted and (b) S K-edge k^2-weighted EXAFS experimental results (open circles) and theoretical fitting (solid lines) of (Bi, S)TiO_2.

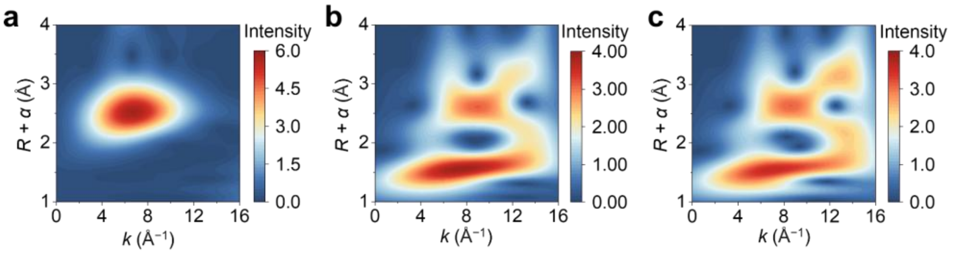

Figure S18. (a–c) Wavelet transform analysis of the k^2-weighted EXAFS data of Ti foil, TiO_2, and (Bi, S)TiO_2.

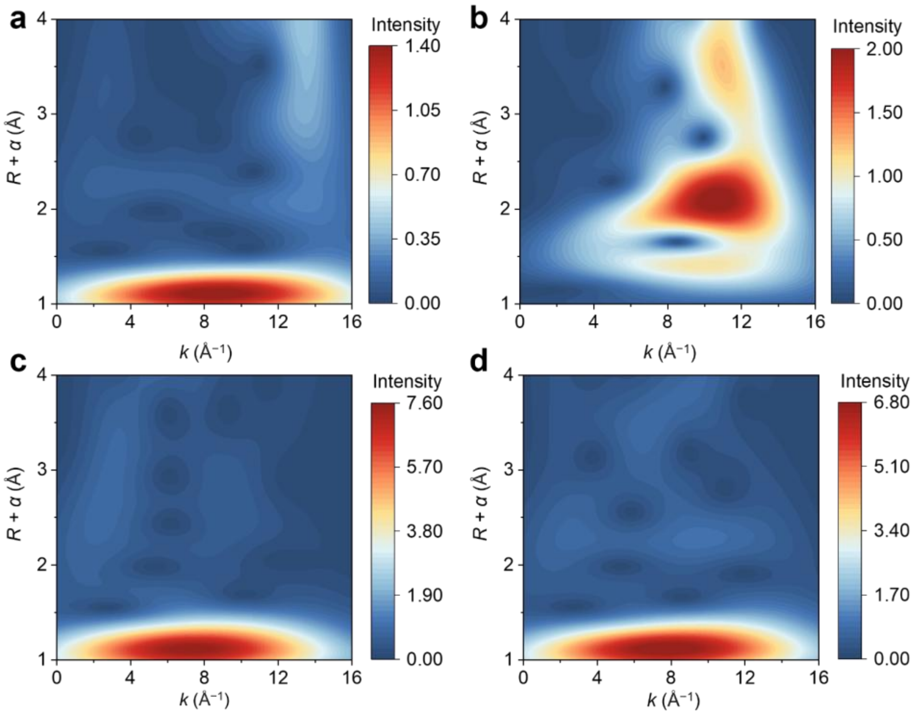

Figure S19. (a–d) Wavelet transform analysis of S element of EXAFS data of (Bi, S)TiO_2, S powder, Na_2SO_3, and Na_2SO_4.

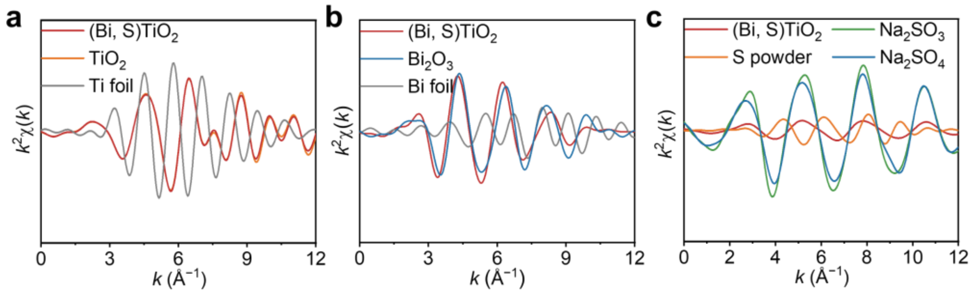

Figure S20. *k* space fitting curve of (a) Ti K-edge, (b) Bi L-edge, and (c) S K-edge.

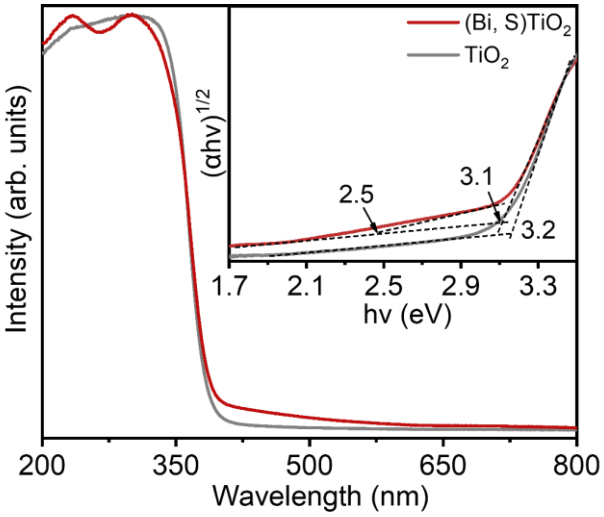

Figure S21. UV-visible light absorption spectra of TiO_2 and (Bi, S)TiO_2.

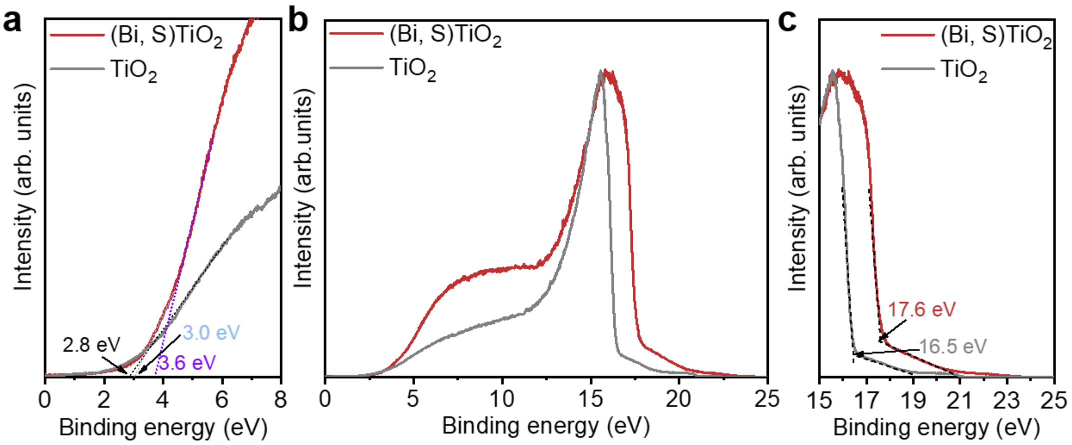

Figure S22. UPS spectra of TiO_2 and (Bi, S)TiO_2.

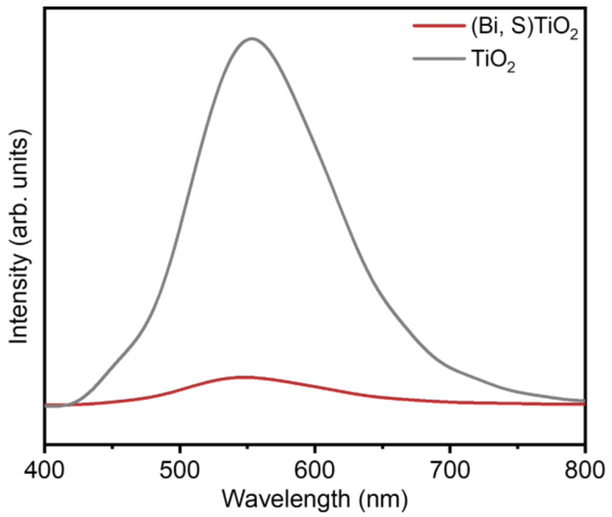

Figure S23. PL spectra of TiO_2 and (Bi, S)TiO_2.

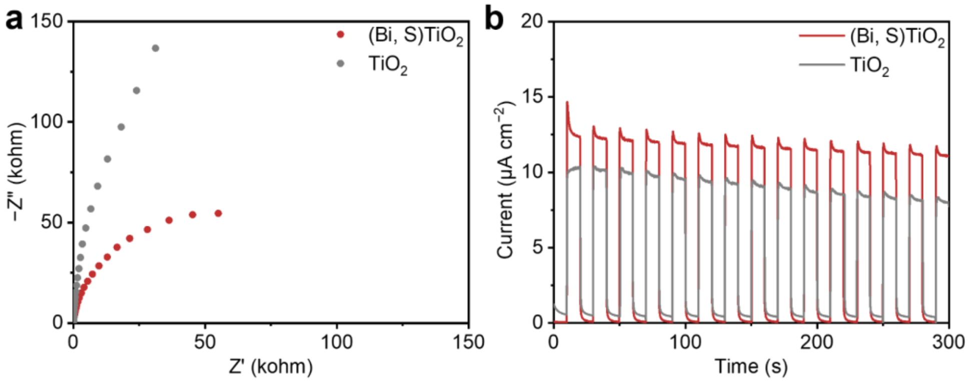

Figure S24. EIS and photocurrent curves of TiO_2 and (Bi, S)TiO_2.

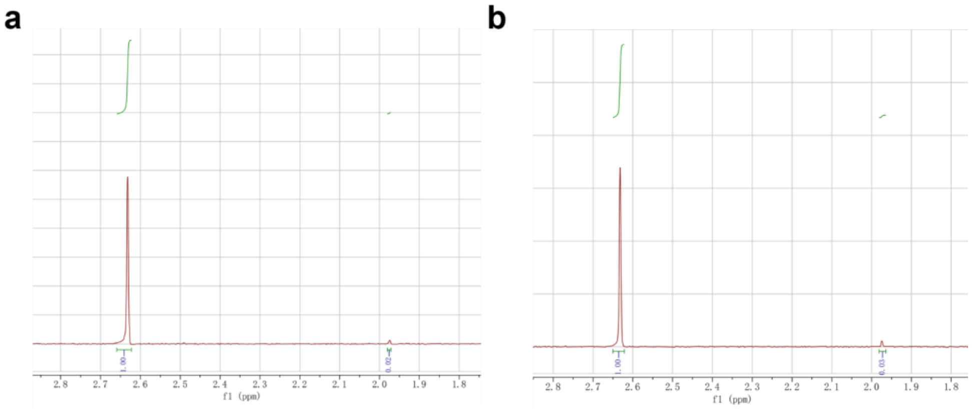

Figure S25. NMR analysis of photocatalytic CO_2 reduction of (Bi, S)TiO_2.

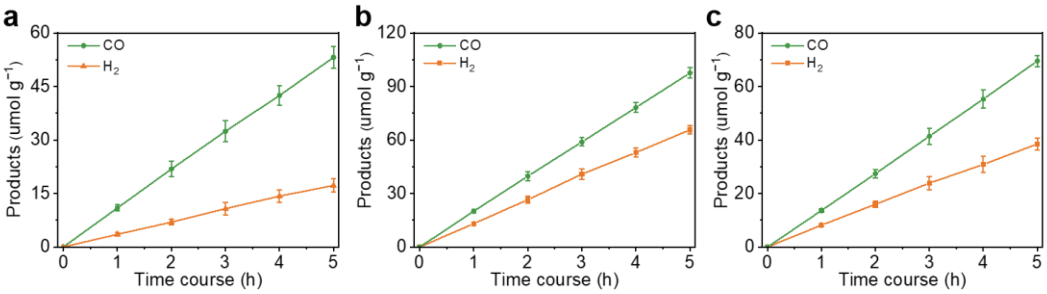

Figure S26. Products analysis of the time-dependent photocatalytic CO_2RR for (a) TiO_2, (b) Bi-TiO_2, and (c) S-TiO_2.

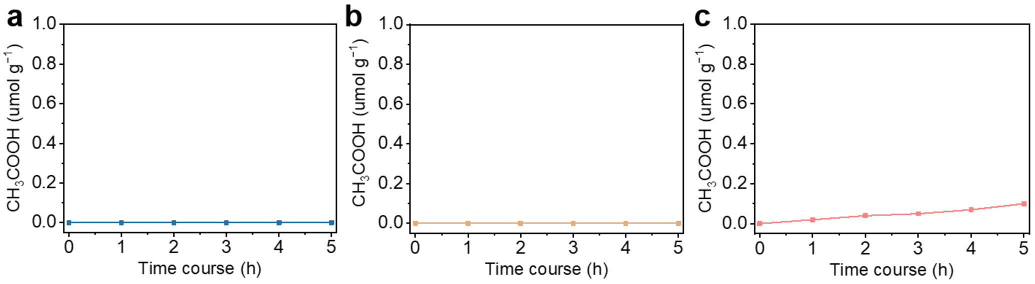

Figure S27. Control experiments on (Bi, S)TiO_2 under three deficient conditions: (a) without irradiation, (b) without photocatalyst, and (c) without Na_2SO_3 for photocatalytic CO_2 reduction to acetic acid.

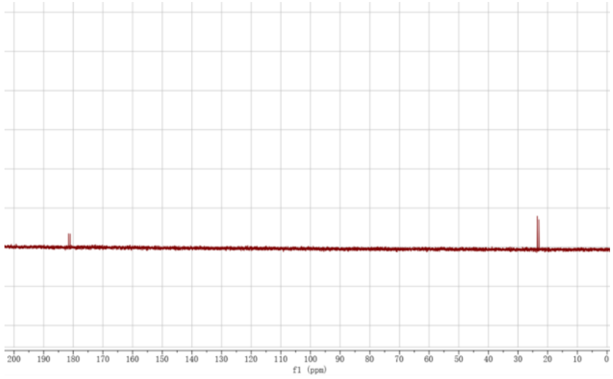

Figure S28. The photocatalytic ^{13}C-labeled CO_2 reduction to acetic acid of (Bi, S)TiO_2 under UV-vis light and Na_2SO_3 as sacrificial agent.

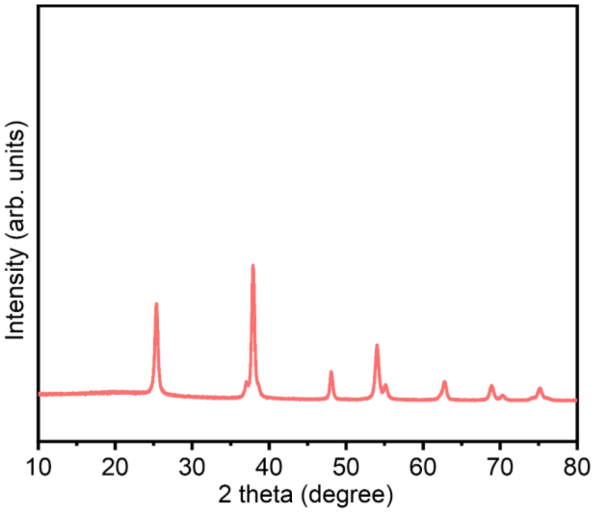

Figure S29. The post-reaction XRD pattern of (Bi, S)TiO_2 after long-term CO_2RR.

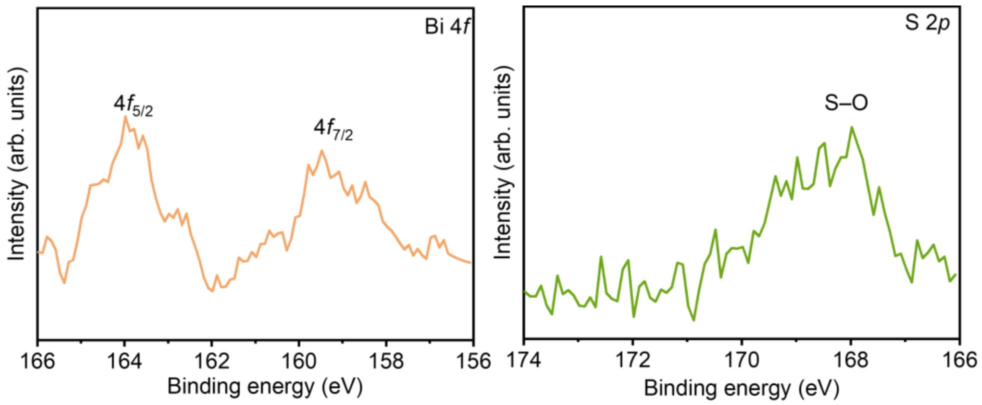

Figure S30. High-resolution XPS spectra of Bi 4*f* and S 2*p* XPS for (Bi, S)TiO_2 after long-term CO_2RR.

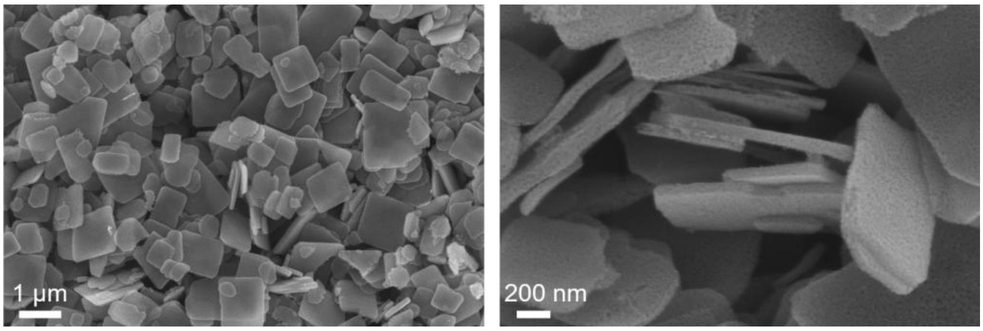

Figure S31. The SEM images of (Bi, S)TiO_2 after long-term CO_2RR.

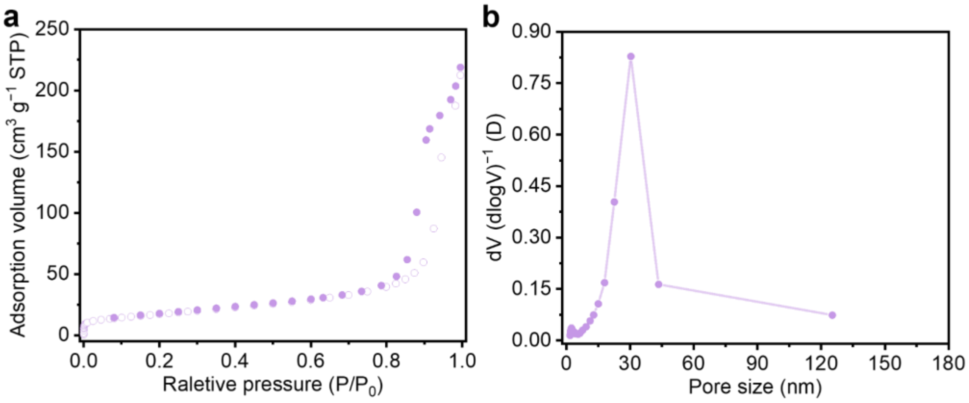

Figure S32. Adsorption–desorption isotherms (a) and pore size distribution (b) of (Bi, S)TiO_2 after long-term CO_2RR with specific surface area of 63.01 m^2 g^{-1}.

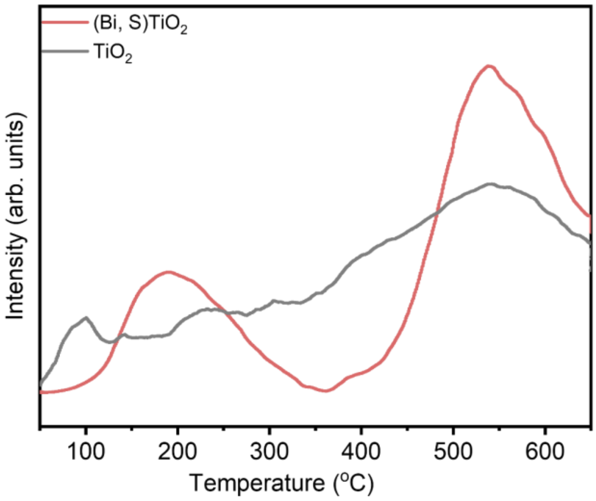

Figure S33. CO_2-TPD signal analysis of (Bi, S)TiO_2 and TiO_2.

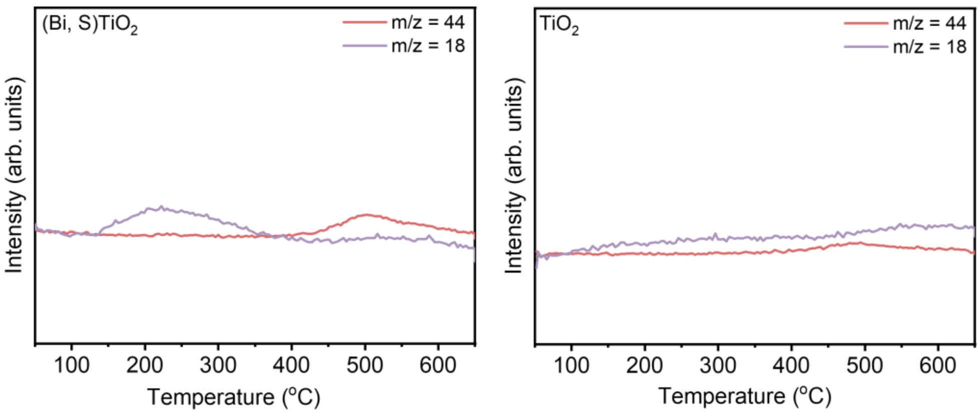

Figure S34.TPD-Mass of (Bi, S)TiO_2 and TiO_2 in CO_2 atmosphere.

Tables

Table S1. The element content of (Bi, S)TiO_2 and TiO_2 from ICP-OES.

Sample	Ti (at%)	O (at%)	S (at%)	Bi (at%)
**(Bi, S)TiO_2**	29.80	67.78	1.40	1.02
**TiO_2**	30.79	69.21	0	0

Table S2. EXAFS curves fitting parameters of Bi L-edge and S K-edge.

Path	^{a}N	^{b}R [Å]	Bond disorder $^{c}\sigma^2$ [× 10^{-3}Å^2]	$^{d}\Delta E_0$ [eV]	R-factor (%)
**S–O**	1.6	1.45	3	3.5	2.5
**Bi–O1**	1.8	2.11	0.2	−2.3	2
**Bi–O2**	1.9	2.26	5		

^{a}N: coordination numbers; ^{b}R: bond distance; $^{c}\sigma^2$: Debye-Waller factors; $^{d}\Delta E_0$: the inner potential correction. R-factor: goodness of fit.

Table S3. The energy band of (Bi, S)TiO_2 and TiO_2.

**Samples**	**Bandgap (V vs. NHE)**	**VB (V vs. NHE)**	**CB (V vs. NHE)**
**(Bi, S)TiO_2**	3.1/2.5	2.7/2.1	−0.4
**TiO_2**	3.2	3	−0.2

Table S4. TA spectra carriers' kinetics and fitting parameters.

Samples	A_1 (× 10^{-3})	τ_1 (ps)	A_2 (× 10^{-3})	τ_2 (ps)	τ_{ave} (ps)
**(Bi, S)TiO_2 (650 nm)**	1.41	231.1	2.13	4538.4	4397.9
**TiO_2 (650 nm)**	0.62	147.9	1.76	1686.2	1640.4

Table S5. The parameters of TRPL of (Bi, S)TiO_2 and TiO_2.

**Sample**	**τ_1 (ns)**	**A_1**	**τ_2 (ns)**	**A_2**	**τ_{ave} (ns)**
**TiO_2**	81	24%	569	76%	452
**(Bi, S)TiO_2**	85	23%	635	77%	509

Table S6. Comparison of photocatalytic CO_2 reduction performance to C_2 products with those of reported photocatalysts.

Materials	Light source	Multiphase system	C_2 yield rate (μmol h^{-1} g^{-1})	Products of C_2	Selectivity	Ref.
**(Bi, S)TiO_2**	Xenon lamp	Gas-liquid-solid	66.7	Acetate	89	This work
**UiO-66/MoS_2**	Xenon lamp, > 400 nm	Gas-solid	39	Acetic acid	94	[6]
**Au_n/Au_1–MoS_2**	Xenon lamp	Gas-liquid-solid	28.7	Ethanol, acetate	95.1	[7]
**Pd–Nb_2O_5**	Xenon lamp, AM 1.5G filter	Gas-solid	15.3	Acetic acid, ethylene	44.4	[8]
**WO_3·0.33H_2O**	Solar simulator	Gas-liquid-solid	9.4	Acetate	85	[9]
**In_2S_3/C/Fe_3C**	Xe lamp, AM 1.5 G	Gas-liquid-solid	11.9	Ethanol, acetate	93	[10]
**PQD@PCN-6**	Xenon lamp	Gas-solid	16.3	Ethane	20	[11]
**CuO_x@p-ZnO**	Xenon lamp, 320–780 nm	Gas-solid	2.3	Ethylene	32.9	[12]
**Pt–G/RBT**	Solar simulator	Gas-solid	6.7	Ethane	27.9	[13]
**$Cu^{\delta+}$/CeO_2-TiO_2**	Xenon lamp	Gas-liquid-solid	4.5	Ethylene	73.9	[14]
**Vo-rich Zn_2GeO_4**	Xenon lamp, AM 1.5G filter	Gas-solid	12.7	Acetic acid	66.9	[15]
**Magnetic-field-TiO_2**	UV-enhanced Xenon light	Gas-liquid-solid	6.2	Ethanol	66.7	[16]
**Cu SAs/UiO-66-NH_2**	Xenon lamp, > 400 nm	Gas-liquid-solid	4.2	Ethanol	44.2	[17]
**Cu/GO**	Halogen lamp	Gas-solid	3.9	Acetaldehyde	56.9	[18]
**CuACs/PCN**	Xenon lamp	Gas-liquid-solid	10.2	Ethylene	53.2	[19]
**Sv-CdS@ZIF-8**	Xenon lamp, > 420 nm	Gas-Solid	0.8	Ethylene	12.8	[20]
**Au–Pd/TiO_2**	Xenon lamp	Gas-solid	1.5	Ethylene, ethane	14	[21]
**NiV_2Se_4**	Xenon lamp	Gas-solid	1.9	Ethane	77.2	[22]
**InCu/PCN**	Xenon lamp	Gas-liquid-solid	28.5	Ethanol	92	[23]
**TJU-32**	Xenon lamp	Gas-liquid-solid	50.5	Ethanol	89.4	[24]

Table S7. The element content of (Bi, S)TiO_2 after CO_2RR reaction from ICP-OES.

Sample	Ti (at%)	O (at%)	S (at%)	Bi (at%)
**(Bi, S)TiO_2**	29.70	67.85	1.42	1.03

Supporting References

[1] S. J. Clark, M. D. Segall, C. J. Pickard, P. J. Hasnip, M. I. J. Probert, K. Refson, M. C. Payne, *Z. Kristallogr. - Cryst. Mater.* **2005**, *220*, 567.

[2] J. P. Perdew, K. Burke, M. Ernzerhof, *Phys. Rev. Lett.* **1996**, *77*, 3865.

[3] P. J. Hasnip, C. J. Pickard, *Comput. Phys. Commun.* **2006**, *174*, 24.

[4] J. P. Perdew, J. A. Chevary, S. H. Vosko, K. A. Jackson, M. R. Pederson, D. J. Singh, C. Fiolhais, *Phys. Rev. B* **1992**, *46*, 6671.

[5] J. D. Head, M. C. Zerner, *Chem. Phys. Lett.* **1985**, *122*, 264.

[6] F. Yu, X. Jing, Y. Wang, M. Sun, C. Duan, *Angew. Chem. Int. Ed.* **2021**, *60*, 24849.

[7] C. Chen, C. Ye, X. Zhao, Y. Zhang, R. Li, Q. Zhang, H. Zhang, Y. Wu, *Nat. Commun.* **2024**, *15*, 7825.

[8] J. Ding, P. Du, P. Li, W. Liu, J. Xu, W. Yan, Y. Pan, J. Hu, J. Zhu, Q. Chen, X. Jiao, Y. Xie, *Angew. Chem. Int. Ed.* **2025**, *64*, e202414453.

[9] S. Sun, M. Watanabe, J. Wu, Q. An, T. Ishihara, *J. Am. Chem. Soc.* **2018**, *140*, 6474.

[10] C. Liao, W. Jing, F. Wang, Y. Liu, *Materials Today Catalysis* **2023**, *3*, 100030.

[11] J. Xu, M. Chong, W. Li, E. Zhu, H. Jin, L. Liu, Y. Ren, Y. Zhu, *Chem* **2025**, *11*.

[12] W. Wang, C. Deng, S. Xie, Y. Li, W. Zhang, H. Sheng, C. Chen, J. Zhao, *J. Am. Chem. Soc.* **2021**, *143*, 2984.

[13] S. Sorcar, J. Thompson, Y. Hwang, Y. H. Park, T. Majima, C. A. Grimes, J. R. Durrant, S.-I. In, *Energy Environ. Sci.* **2018**, *11*, 3183.

[14] T. Wang, L. Chen, C. Chen, M. Huang, Y. Huang, S. Liu, B. Li, *ACS Nano* **2022**, *16*, 2306.

[15] J. Zhu, W. Shao, X. Li, X. Jiao, J. Zhu, Y. Sun, Y. Xie, *J. Am. Chem. Soc.* **2021**, *143*, 18233.

[16] M.-P. Jiang, K.-K. Huang, J.-H. Liu, D. Wang, Y. Wang, X. Wang, Z.-D. Li, X.-Y. Wang, Z.-B. Geng, X.-Y. Hou, S.-H. Feng, *Chem* **2020**, *6*, 2335.

[17] G. Wang, C.-T. He, R. Huang, J. Mao, D. Wang, Y. Li, *J. Am. Chem. Soc.* **2020**, *142*, 19339.

[18] I. Shown, H.-C. Hsu, Y.-C. Chang, C.-H. Lin, P. K. Roy, A. Ganguly, C.-H. Wang, J.-K. Chang, C.-I. Wu, L.-C. Chen, K.-H. Chen, *Nano Lett.* **2014**, *14*, 6097.

[19] W. Xie, K. Li, X.-H. Liu, X. Zhang, H. Huang, *Adv. Mater.* **2023**, *35*, 2208132.

[20] F. Tian, H. Zhang, S. Liu, T. Wu, J. Yu, D. Wang, X. Jin, C. Peng, *Appl. Catal. B* **2021**, *285*, 119834.

[21] Q. Chen, X. Chen, M. Fang, J. Chen, Y. Li, Z. Xie, Q. Kuang, L. Zheng, *Journal of Materials Chemistry A* **2019**, *7*, 1334.

[22] Y. Tian, R. Wang, S. Deng, Y. Tao, W. Dai, Q. Zheng, C. Huang, C. Xie, Q. Zeng, J. Lin, H. Chen, *Nano Lett.* **2023**, *23*, 10914.

[23] H. Shi, H. Wang, Y. Zhou, J. Li, P. Zhai, X. Li, G. G. Gurzadyan, J. Hou, H. Yang, X. Guo, *Angew. Chem. Int. Ed.* **2022**, *61*, e202208904.

[24] J. Yin, X. Song, C. Sun, Y. Jiang, Y. He, H. Fei, *Angew. Chem. Int. Ed.* **2024**, *63*, e202316080.
